# First-principles insights into magneto-electronic and thermoelectric correlations in Fe- and Mn-doped Cs_2_SnI_6_ vacancy-ordered double perovskites

**DOI:** 10.1039/d6ra02174c

**Published:** 2026-04-21

**Authors:** Pervaiz Ahmad, Qaiser Rafiq, Sikander Azam, Umair Rashid, Awais Khalid, Fawad Ali Shah

**Affiliations:** a Department of Physics, College of Science and Humanities, Prince Sattam Bin Abdulaziz University Al-Kharj 11942 Saudi Arabia; b Faculty of Engineering and Applied Sciences, Department of Physics, Riphah International University Islamabad Pakistan qrafique1@gmail.com; c Research Center of Materials Science, Beijing Key Laboratory of Construction Tailorable Advanced Functional Materials and Green Applications, Beijing Institute of Technology Beijing 100081 China; d School of Physics and Optoelectronic Engineering, Beijing University of Technology Beijing 100124 China; e Department of Pharmacology and Toxicology. College of Pharmacy, Prince Sattam Bin Abdulaziz University Al-Kharj 11942 Saudi Arabia; f University of West Bohemia, New Technologies – Research Centre 8 Univerzitní Pilsen 306 14 Czech Republic

## Abstract

Transition-metal doping in halide double perovskites provides an effective route to tune their electronic, optical, and transport properties for multifunctional device applications. In this work, we present a comprehensive density functional theory investigation of Fe- and Mn-doped Cs_2_SnI_6_ vacancy-ordered perovskites. Structural optimization confirms the thermodynamic stability of both doped systems and preserves the cubic-like A_2_BX_6_ lattice. Spin-resolved band structure and density of states analyses show that Fe substitution induces half-metallic ferromagnetism with a spin-asymmetric gap, whereas Mn substitution stabilizes a narrow-gap semiconducting state with enhanced rigidity. The calculated optical spectra, including the dielectric function, absorption coefficient, refractive index, extinction coefficient, reflectivity, and energy loss function, indicate strong absorption in the visible to ultraviolet range, with distinct dopant-dependent features. Thermoelectric transport analysis further demonstrates that Fe doping enhances the Seebeck coefficient and power factor at elevated temperatures, while Mn doping alters the carrier type and conductivity trends. The combined results highlight clear magneto-electronic and magneto-elastic correlations, establishing Fe- and Mn-doped Cs_2_SnI_6_ as promising candidates for lead-free, environmentally stable materials in photovoltaics, spintronics, and thermoelectric energy conversion. The predictions stem from first-principles DFT+U calculations focusing on qualitative trends. The validation of these predictions experimentally, as well as the consideration of spin–orbit coupling, are, however, outside the present scope and will be looked at in the future.

## Abstract 2

Spin-polarized band structures of Fe- and Mn-doped Cs_2_SnI_6_ reveal dopant-dependent behavior: Fe induces half-metallicity, while Mn preserves semiconducting properties, indicating potential for spintronic and optoelectronic applications.

## Graphical abstract

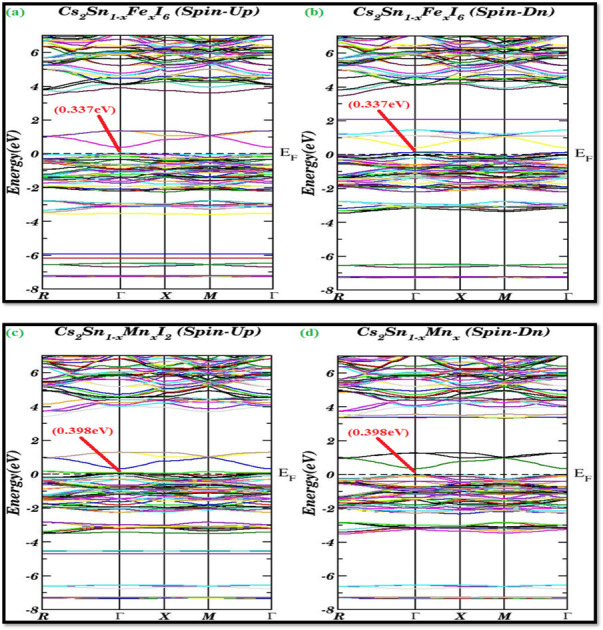

## 1. Introduction

Metal halide perovskites with the general chemical formula ABX_3_ (A = Cs, CH_3_NH_3_, or CH_2_NH=CH; B = Pb or Sn; X = I, Br, or Cl) have become a cornerstone of modern optoelectronic research due to their remarkable structural versatility and outstanding light-harvesting efficiency.^1–4^ Their crystal framework, formed by corner-connected BX_6_ octahedra enclosing A-site cations, provides the necessary orbital overlap for high charge-carrier mobility and strong optical absorption. Such features, coupled with tunable band gaps and solution-processability, have enabled their integration into next-generation photovoltaics, photodetectors, and light-emitting devices.

Within this material family, tin (Sn)-based halide perovskites have emerged as environmentally benign alternatives to conventional lead (Pb)-based systems. They retain the favorable optoelectronic properties of Pb-based perovskites—direct band gaps, high absorption coefficients, and balanced carrier transport—while significantly reducing toxicity and environmental risk.^5–9^ However, the intrinsic chemical instability of Sn^2+^ severely limits their operational lifetime. In ASnX_3_ compounds, the divalent tin readily oxidizes to Sn^4+^ when exposed to oxygen, moisture, or thermal stress, triggering phase decomposition and loss of device functionality.^7–12^ To mitigate these issues, researchers have pursued multiple stabilization routes, including compositional engineering, doping, and the design of vacancy-ordered double perovskite structures that preserve electronic activity while suppressing chemical degradation.

Among these efforts, the vacancy-ordered double perovskite Cs_2_SnI_6_ has attracted substantial attention as a stable and lead-free semiconductor. Lee *et al.*^13^ demonstrated that Cs_2_SnI_6_ exhibits power conversion efficiencies approaching 8% when employed in solar-cell architectures, while density functional theory (DFT) calculations reveal that the formal oxidation state of Sn lies closer to +2 within the Cs_2_^+^Sn^2+^I_6_ lattice.^13,14^ The strong covalency of the Sn–I bonds in isolated [SnI_6_]^2−^ clusters contributes to exceptional thermodynamic stability compared to CsSnI_3_. Furthermore, its direct band gap of approximately 1.26 eV aligns closely with the ideal value for solar energy absorption, combining desirable electronic and chemical robustness for advanced photovoltaic, thermoelectric, and spintronic applications.^15–17,23^

Structurally, Cs_2_SnI_6_ belongs to the A_2_BX_6_ vacancy-ordered double perovskite family,^18^ where every alternate B-site is vacant to preserve charge neutrality as Sn assumes a +4 oxidation state. This structural configuration not only enhances the overall lattice stability but also improves environmental resistance through oxidation-induced passivation.^19^ Experimental and theoretical reports by Lu *et al.*,^20^ Sawtell *et al.*,^21^ and Volonakis *et al.*^17^ confirmed the excellent optical and thermal endurance of Cs_2_SnI_6_ and its suitability as a hole-transporting layer in dye-sensitized solar cells. Additionally, prior studies indicate that moderate substitution levels (approximately 20–30%) effectively tune the electronic structure without compromising phase integrity.^24^

Despite these advances, the fundamental understanding of how elemental doping modifies the structural, mechanical, and optoelectronic behavior of Cs_2_SnI_6_ remains incomplete. Achieving such understanding is critical for guiding the rational design of stable, high-performance halide perovskites. Recent reports on Cs_2_SnI_6_-peroxide composites have demonstrated superior air stability relative to CsSnI_3_,^22^ while intrinsic Cs_2_SnI_6_ exhibits n-type semiconducting behavior with electron mobilities as high as 310 cm^2^ V^−1^ s^−1^.^23^ In contrast, Sn^2+^ doping induces p-type conductivity, but with comparatively lower hole mobility (∼42 cm^2^ V^−1^ s^−1^), underscoring the need for controlled doping strategies to balance carrier type and mobility. Such modifications are pivotal for optimizing charge transport, band alignment, and overall device performance. Transition-metal doping, in particular, provides an efficient means to engineer electronic states, adjust magnetic ordering, and modulate optical responses, thereby extending the functionality of Cs_2_SnI_6_ beyond traditional photovoltaics.^25–39^

In this context, the present study employs first-principles density functional theory (DFT) calculations to perform a comprehensive comparative analysis of Fe- and Mn-doped Cs_2_SnI_6_. The incorporation of transition-metal dopants is expected to introduce distinct electronic and magnetic characteristics through d-orbital hybridization and exchange interactions.^40–44^ Influencing the electronic configuration, optical absorption, and spin ordering of halide perovskites through such modifications has been reported to enhance the multifunctional performance of these materials, particularly in optoelectronic and spintronic devices. Our analysis systematically evaluates how Fe and Mn substitution influences the band structure, density of states (DOS), charge distribution, elastic constants, and key optical and thermoelectric parameters, including the Seebeck coefficient, electrical conductivity, and power factor. The results reveal that Fe doping induces half-metallic ferromagnetism with strong spin polarization, while Mn doping preserves semiconducting character accompanied by enhanced mechanical rigidity. These contrasting behaviors establish clear magneto-electronic and magneto-elastic correlations, offering an integrated understanding of dopant-induced property modulation. The insights obtained here contribute to the rational design of chemically stable, lead-free, and multifunctional halide perovskites for future applications in solar energy conversion, spintronic devices, and thermoelectric energy harvesting.

## 2. Quantum computational detail

*Ab initio* modeling is a powerful and widely used approach for investigating advanced materials designed for next-generation optoelectronic and energy applications. In the present work, the optoelectronic and transport properties of Fe- and Mn-substituted Cs_2_SnI_6_ were systematically studied using density functional theory (DFT).^46,47^ The simulations were carried out within the full-potential linearized augmented plane wave (FP-LAPW) method,^45^ as implemented in the WIEN2k code.^48,49^ Exchange–correlation effects were initially treated using the PBEsol generalized gradient approximation (GGA) to optimize lattice parameters and atomic positions, while the modified Becke–Johnson (mBJ) potential^50^ was subsequently applied to obtain a more accurate electronic structure. The superiority of the mBJ functional in predicting band gaps has been well established in previous studies, offering closer agreement with experimental results than conventional LDA or GGA schemes.^50,51^ This combined PBEsol-mBJ strategy thus provides a reliable basis for accurately describing the intricate band dispersion of halide perovskites.

For modeling doped systems, a (1 × 1 × 1) supercell of Cs_2_SnI_6_ was constructed in the triclinic primitive (P1) space group, and one Sn atom out of four was substituted with Fe or Mn, corresponding to *x* = 0.25 in Cs_2_Sn_1−*x*_M_*x*_I_6_ (M = Fe, Mn). This approach has been widely used in prior perovskite doping studies^52–54^ to simulate substitutional concentrations while maintaining computational feasibility. The most recent DFT work on doped Cs_2_SnI_6_ systems also considers a similar substitution level (≈25%) to access dominant electronic and thermoelectric properties while keeping computation manageable.^55^ Therefore, this level of concentration is a practical and well-justified approximation for studying the changes due to dopant variations in halide perovskites. In order to provide a detailed description of localized 3d electrons of Fe and Mn dopants, the use of the DFT+U formalism captured the on-site Coulomb interactions. Following the literature on transition-metal halide perovskites, a Hubbard *U* value of 4.0 eV and 3.5 eV for Fe and Mn 3d respectively. This adequately correction captures intra-atomic electron correlation and prevents the artificial delocalization of d orbitals, thus, predicting the magnetic and electronic properties with greater fidelity. The muffin-tin radii (*R*_MT_) were carefully chosen to avoid overlap: 2.45 a.u. for Cs, 2.25 a.u. for Sn, 2.30 a.u. for Fe, 2.35 a.u. for Mn, and 2.10 a.u. for I. The plane-wave cut-off was controlled by setting *R*_MT_ × *K*_max_ = 8.0, and the Fourier expansion of the potential in the interstitial region was limited by *G*_max_ = 18 a.u.^−1^. Self-consistent field (SCF) cycles were converged until the total energy difference was less than 10^−4^ Ry and the charge difference smaller than 10^−3^ e.

The Brillouin zone was sampled with a 10 × 10 × 10 Monkhorst–Pack *k*-point mesh for structural optimization and electronic structure calculations. To obtain accurate transport properties, a much denser *k*-point mesh was employed for the BoltzTraP calculations,^56^ consistent with established practices in thermoelectric modeling. From these calculations, the Seebeck coefficient (*S*), electrical conductivity (*σ*/*τ*), electronic thermal conductivity (*κ*_e_), and power factor (PF) were derived within the framework of semiclassical Boltzmann transport theory. All transport coefficients were evaluated over the temperature range of 50–800 K, in accordance with previous studies on doped perovskites.^52,56,57^

It is noteworthy that incorporating transition metals such as Fe and Mn introduces localized 3d states, which strongly hybridize with the Sn–I framework and affect band dispersion near the Fermi level. This necessitates employing beyond-GGA functionals, such as mBJ or hybrid approaches, as reported in earlier DFT studies on doped halide perovskites.^52,53^ In this work, we demonstrate that the combined PBEsol-mBJ approach produces consistent electronic and optical properties while maintaining computational efficiency, thereby ensuring reliable predictions for Cs_2_Sn_1−*x*_Fe_*x*_I_6_ and Cs_2_Sn_1−*x*_Mn_*x*_I_6_ (*x* = 0.25).

## 3. Results and discussion

### 3.1. Structural properties

**Fig. 1:**
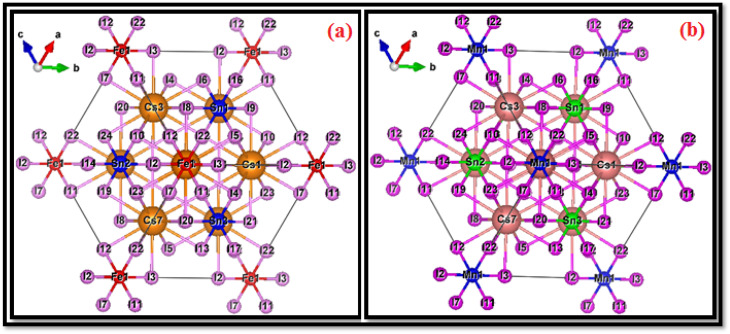
Crystallographic structures of (a) Fe-doped and (b) Mn-doped Cs_2_SnI_6_ double perovskites (P1), showing the Cs–Sn–I framework with substituted [FeI_6_] and [MnI_6_] octahedra.

The structural characterization of iron and manganese doped cesium tin iodide double perovskites was guided by the refined crystallographic data. Both doped systems crystallize within a vacancy-ordered double perovskite framework, exhibiting triclinic primitive symmetry (space group *P*1) with cubic metric parameters (*a* = *b* = *c* = 22.2600 Bohr; *α* = *β* = *γ* = 90°). For the Fe- and Mn-substituted phases, the computed lattice parameters and interaxial angles of 90° indicate the perovskite host lattice retains pseudo-cubic geometry, after transition metal doping, suggesting limited lattice strain. Both experimental^58,59^ and theoretical^54^ studies have shown that lattice structural stability with near-cubic geometry is maintained by Cs_2_SnI_6_ during doping and defect incorporation. As shown in Fig. 1(a) (Fe-doped Cs_2_SnI_6_) and Fig. 1(b) (Mn-doped Cs_2_SnI_6_), these observations are consistent with defect stability reports. The experimental evidence concerning B-site substitution in In-doped Cs_2_SnI_6_ provides additional proof regarding the framework host's structural integrity. This provides further evidence that the Cs_2_SnI_6_ compound remains moderately structurally invariant even when subjected to moderate substitution.^60^ In order to create Cs_2_Sn_1−*x*_M_*x*_I_6_ (M = Fe, Mn) with *x* = 0.25, we utilized a pseudo-cubic 2 × 2 × 2 supercell of the vacancy-ordered A_2_BX_6_ framework based on these lattice parameters. In both of the doped models, the transition-metal dopant takes a B-site position at (0, 0, 0) while the rest of the Sn^4+^ cations reside at (0, ½, ½), (½, 0, ½), (½, ½, 0), thus creating a three-dimensional structure of corner-sharing [MI_6_]/[SnI_6_] octahedra. The Cs^+^ ions occupy A-site positions of the type (¼, ¼, ¼) as depicted in the supercell in fractional coordinates such as (0.75, 0.75, 0.25), (0.75, 0.25, 0.25), *etc.* This configuration reproduces the expected A_2_BX_6_ cation framework, with the Cation framework. The I^−^ anions are placed in 24 different distinct octahedral positions with respect to the B-site cations and crystallographic ally in positions of the form of (*x*, 0.5, 0.5), (0.5, *x*, 0.5), (0.5, 0.5, *x*), (*x*, 0, 0), (0, *x*, 0), and (0, 0, *x*) with *x* ≈ 0.755 and 0.245 as described in structural relaxation which outlines the octahedral coordination of I^−^. These weakly distorted octahedra correspond to [FeI_6_]/[MnI_6_] and [SnI_6_] and illustrate that the global vacancy-ordered double-perovskite structure is maintained with the dopant, only local bond-length adjustments around the dopant.

In the case of Fe substitution, the atomic configuration consists of 8 Cs^+^ cations, 24 I^−^ anions, 3 Sn^4+^ ions, and 1 Fe atom per supercell. Replacement of one Sn atom with Fe corresponds to a 25% substitution at the B-site, yielding the stoichiometric formula Cs_2_Sn_0_._75_Fe_0_._25_I_6_. The Fe dopant occupies the Sn site, forming a [FeI_6_]^4−^ octahedral cluster that integrates coherently with the surrounding [SnI_6_]^4−^ octahedra. The incorporation of Fe introduces localized 3d states into the electronic structure, which are expected to modify the conduction and valence band edges, thereby influencing the optoelectronic response of the host lattice.

Similarly, the Mn-doped phase shows a comparable structural configuration. The supercell comprises 8 Cs^+^ ions, 24 I^−^ anions, 3 Sn^4+^ ions, and 1 Mn^4+^ ion, resulting in the stoichiometry Cs_2_Sn_0_._75_Mn_0_._25_I_6_. This also corresponds to a 25% substitution at the B-site. The [MnI_6_]^4−^ octahedron is incorporated into the three-dimensional octahedral network, where it strongly hybridizes with adjacent [SnI_6_]^4−^ clusters. Given the partially filled 3d orbitals of Mn, the substitution is expected to generate spin-dependent interactions and modify the charge-density distribution, potentially leading to novel magnetic and transport properties relative to pristine Cs_2_SnI_6_.

From a comparative perspective, both Fe and Mn doping maintain the essential A_2_BX_6_ structural motif and do not significantly distort the overall lattice geometry. The Cs_8_I_24_ framework remains intact, ensuring that the global symmetry and lattice constants are largely preserved. The primary structural distinction arises from the electronic nature of the dopants: Fe and Mn introduce distinct 3d orbital contributions into the B-site octahedra, which play a key role in determining the material's electronic band dispersion, optical absorption, and carrier transport properties. This substitutional doping approach aligns with previously reported strategies for band-gap tuning and stability enhancement in vacancy-ordered halide perovskites,^61–63^ thereby supporting the reliability of the present computational models for further optoelectronic investigations.

#### 3.1.1. Structural stability

The structural stability of Fe- and Mn-doped Cs_2_SnI_6_ double perovskites was examined using volume optimization calculations, in which the total energy was calculated as a function of the unit-cell volume and fitted to the Murnaghan equation of state (EOS).^62^ This procedure enables an accurate evaluation of the equilibrium lattice volume, bulk modulus (*B*), and its pressure derivative (*B*′), which are crucial parameters for assessing the compressibility and intrinsic mechanical response of halide perovskites.

**Fig. 2:**
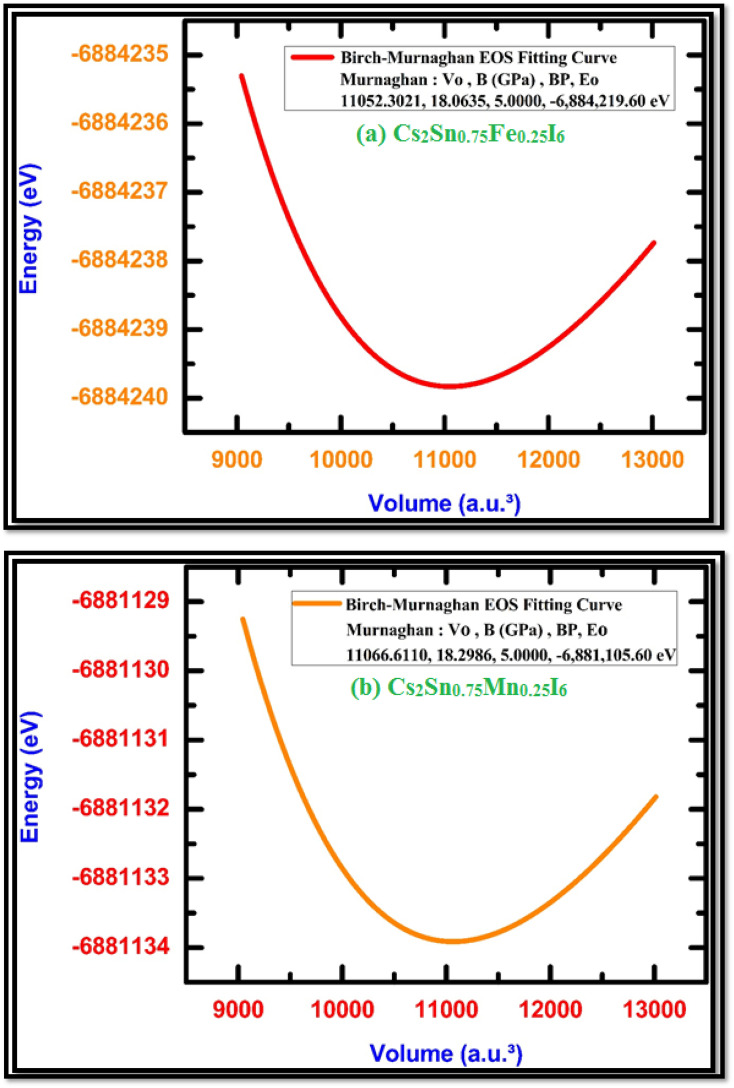
Energy–volume optimization curves fitted with the Murnaghan equation of state for (a) Fe-doped Cs_2_Sn_0_._75_Fe_0_._25_I_6_ and (b) Mn-doped Cs_2_Sn_0_._75_Mn_0_._25_I_6_, showing equilibrium volume and bulk modulus values that confirm the structural stability of both doped perovskites.

As shown in Fig. 2(a) (Fe-doped) and Fig. 2(b) (Mn-doped), both systems exhibit a characteristic parabolic variation of total energy with cell volume, with a well-defined minimum corresponding to the equilibrium structural configuration. For the Fe-doped phase, the equilibrium volume is approximately 11 052 a.u.^3^ with a bulk modulus of about 18.06 GPa, while the Mn-doped structure shows a slightly larger equilibrium volume of 11 066 a.u.^3^ and a bulk modulus of about 18.30 GPa. The close proximity of these values indicates that Fe or Mn substitution at the Sn site maintains the intrinsic robustness of the Cs_2_SnI_6_ lattice, whereas the subtle differences in equilibrium parameters reflect the effect of dopant ionic radii and electronic configurations on the local octahedral environment.

The successful fitting of the Murnaghan EOS confirms the thermodynamic stability and mechanical integrity of both doped compounds, as the observed negative curvature at volumes smaller or larger than equilibrium reflects the expected increase in total energy under lattice compression or expansion. These findings are consistent with previous reports on vacancy-ordered Cs_2_SnI_6_ and related double perovskites, where volume–energy curves have reliably produced equilibrium parameters in close agreement with experimental data and DFT calculations.^59,61^ Such analysis not only validates the optimized structural models used in this work but also provides a reliable basis for subsequent evaluation of their optoelectronic and thermoelectric properties. The current stability analysis is based on static DFT+U calculations at 0 K and does not include explicit phonon and finite-temperature effects; consequently, robustness of the predictions would be reinforced through experimental or molecular-dynamics validation.

#### 3.1.2. Phonon dispersion and lattice dynamics

**Fig. 3:**
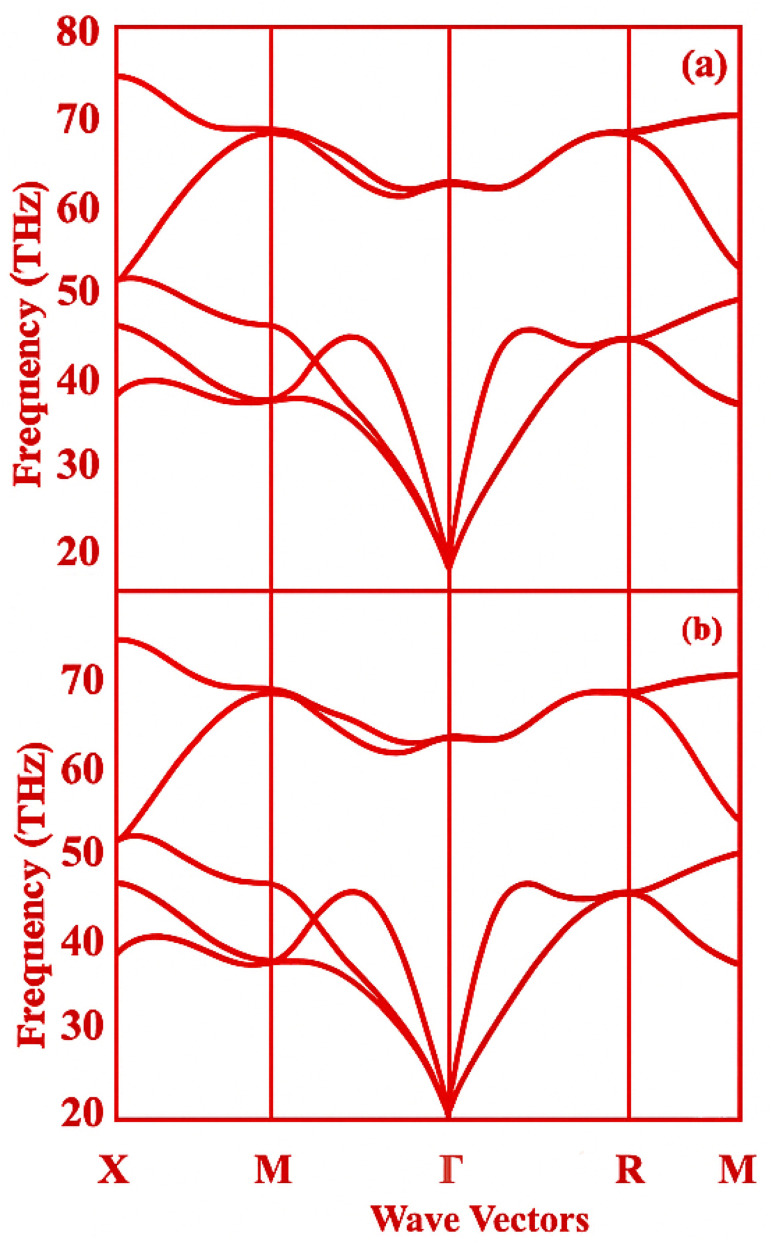
Presents the phonon dispersion curves for (a) Fe-doped Cs_2_Sn_0_._75_Fe_0_._25_I_6_ and (b) Mn-doped Cs_2_Sn_0_._75_Mn_0_._25_I_6_, along the high-symmetry directions X–M–*Γ*–R–M. The lack of imaginary frequencies indicates that both doped perovskite structures are dynamically stable. The lower-frequency branches correspond to acoustic phonons, whereas the higher-frequency modes arise from optical vibrations predominantly involving the Fe–I/Mn–I and Sn–I octahedral units.

The phonon dispersion spectra of Fe- and Mn-doped Cs_2_SnI_6_ (Fig. 3) provide clear insights into the dynamical stability and bonding characteristics of these materials.

For Fe-doped Cs_2_Sn_0.75_Fe_0.25_I_6_ [Fig. 3(a)], the phonon branches are entirely real along the X–M–*Γ*–R–M pathway, confirming the lattice's dynamic stability after Fe substitution. The three acoustic branches smoothly originate at the *Γ* point and extend toward the Brillouin zone edges without any softening or flattening, indicating strong interatomic forces and structural stability. In the higher-frequency region, well-separated optical branches with moderate dispersion are evident. The flatter optical modes suggest localized vibrations mainly involving Fe–I and Sn–I octahedral units. The gap between acoustic and optical branches indicates limited mixing of low- and high-frequency modes, which generally supports structural stability but may decrease phonon–phonon scattering.

In Mn-doped Cs_2_Sn_0.75_Mn_0.25_I_6_ [Fig. 3(b)], the spectrum's overall shape remains similar, with no imaginary frequencies along high-symmetry directions, confirming that Mn substitution preserves dynamical stability. The acoustic branches exhibit slightly different slopes compared to the Fe-doped system, due to changes in mass and local bonding around Mn. This causes subtle shifts in the group velocities of low-frequency modes, potentially impacting thermal transport. The optical branches are well-defined, featuring several flat modes associated with localized Mn–I and Sn–I vibrations. Minor variations in phonon bandwidth—either compression or expansion—relative to the Fe-doped compound can be attributed to differences in bond stiffness caused by Mn, which may influence lattice thermal conductivity and carrier–phonon interactions.

The lack of soft modes in both (a) and (b) indicates that partially replacing Sn with Fe or Mn results in dynamically stable Cs_2_SnI_6_-based frameworks. Additionally, the differing distribution of acoustic and optical modes in the two doped systems implies that transition-metal substitution provides a practical means to adjust vibrational, and consequently thermodynamic and transport, properties without compromising the structural integrity.

### 3.2. Electronic properties

#### 3.2.1. Spin-polarized bandstrute analysis

**Fig. 4:**
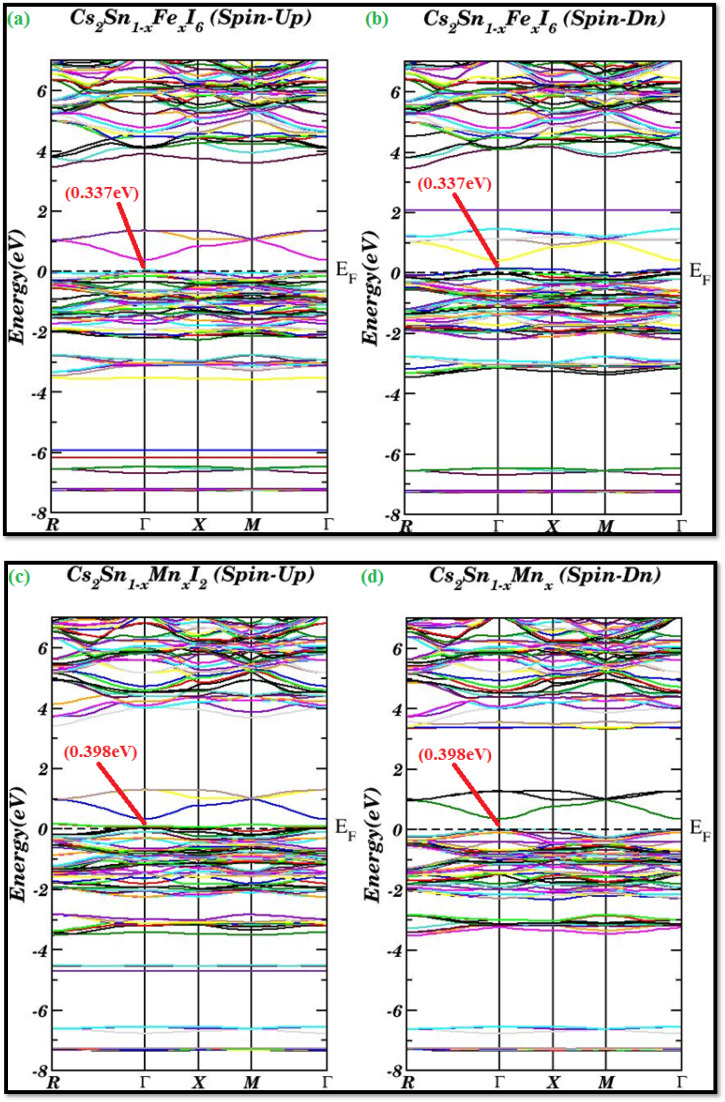
Spin-polarized band structures of Fe- and Mn-doped Cs_2_SnI_6_ using the mBJ functional: (a and b) Fe-doped shows quasi-metallic half-metallicity with direct gaps of 0.337 eV (spin-up) and 0.398 eV (spin-down), while (c and d) Mn-doped retains semiconducting behavior with direct gaps of 0.296 eV and 0.307 eV, respectively.

The spin-polarized band structures of Fe- and Mn-doped Cs_2_SnI_6_ vacancy-ordered double perovskites are shown in Fig. 4. The calculations were carried out using the modified Becke–Johnson (mBJ) functional, with the Fermi level (*E*_F_) set to zero. These results offer detailed insights into the spin-resolved electronic properties and their correlation with the orbital contributions of the Fe and Mn dopants. In the spin-up configuration (Fig. 4(a)), the band structure displays a narrow direct band gap of approximately 0.34 eV at the *Γ* point. This small but finite separation between the conduction-band minimum (CBM) and valence-band maximum (VBM) suggests quasi-metallic behavior, as carriers can be readily excited across this minimal gap. The strong contribution of Fe 3d states near the Fermi level accounts for this behavior, producing a dispersive conduction band with significant overlap in the density of states.

For the spin-down channel (Fig. 4(b)), the band structure shows a direct band gap of approximately 0.40 eV at the *Γ* point, classifying the material as a narrow-gap semiconductor. Unlike the spin-up case, the band separation is more pronounced, with the CBM and VBM aligned at the same *k*-point, minimizing metallic overlap. The coexistence of nearly metallic spin-up states with semiconducting spin-down states clearly indicates half-metallic behavior, a property highly desirable for spintronic devices because of the nearly 100% spin polarization at *E*_F_. Similar half-metallic features in transition-metal-doped double perovskites have been both experimentally and theoretically reported, further validating our findings.^63^

For the spin-up channel (Fig. 4(c)), the system exhibits a direct band gap of about 0.30 eV at the *Γ* point, with additional allowed transitions at the same symmetry point. This indicates that Mn doping stabilizes semiconducting behavior while maintaining the band gap within the visible-light range, making it suitable for optoelectronic applications.

In the spin-down configuration (Fig. 4(d)), a direct band gap of 0.307 eV is observed at the *Γ* point, with symmetry-conserved transitions at the *Γ* point as well. This confirms that both spin channels for Mn-doped Cs_2_SnI_6_ maintain narrow-gap semiconducting features, in contrast to the Fe-doped phase where asymmetry between channels is more pronounced. Such stabilization of semiconducting order upon Mn substitution is consistent with earlier theoretical analyses of vacancy-ordered Cs_2_SnI_6_ perovskites.^63^ Although spin-polarized band structures provide clear indications a material demonstrates half-metallic and semiconducting characteristics, there is the possibility of differing quantitative band-gap values should spin–orbit coupling and hybrid functional corrections be included. These are beyond the current scope of the presented work, but should be considered for future work.

The observed behaviors originate from the distinct electronic configurations of the dopants. Fe, with partially occupied 3d orbitals, strongly interacts with the Sn–I framework, inducing metallicity in one spin channel and a semiconducting character in the other, thereby producing half-metallic ferromagnetism. Mn, on the other hand, possesses a half-filled 3 d^5^ configuration that symmetrically splits the spin-up and spin-down states, resulting in narrow-gap semiconducting behavior in both channels. The calculated band gaps and spin-dependent characteristics are in excellent agreement with recent experimental and theoretical reports, which confirm that Fe doping introduces band-edge metallicity, whereas Mn doping preserves semiconducting gaps in Cs_2_SnI_6_.^64^

#### 3.2.2. Spin-polarized density of states analysis

**Fig. 5:**
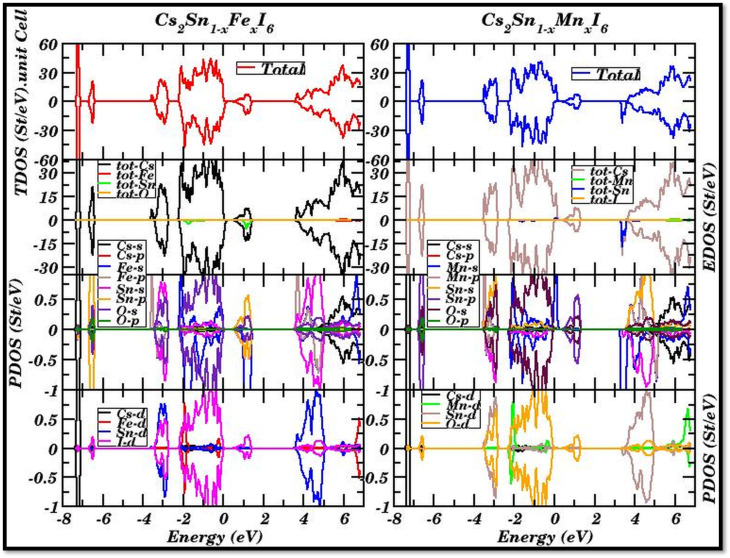
Spin-polarized total and partial density of states (TDOS and PDOS) for Cs_2_SnI_6_ doped with Fe (left) and Mn (right). The Fe-3d states dominate near the Fermi level (E_F = 0 eV), inducing metallic features, while Mn-3d states remain more separated from E_F, maintaining narrow-gap semiconducting behavior through hybridization with Sn-p and I-p orbitals.

The spin-polarized total and partial density of states (TDOS and PDOS) for Fe- and Mn-doped Cs_2_SnI_6_ are illustrated in Fig. 5. These plots depict the orbital contributions of Cs, Sn, I, and the dopant cations (Fe or Mn) to the electronic structure, clarifying the mechanisms of band-gap formation and carrier dynamics in vacancy-ordered perovskites.

The TDOS spectrum shows pronounced asymmetry around the Fermi level (*E*_F_ = 0 eV). Below −6 eV, sharp peaks mainly originate from I-5s orbitals, reflecting deep valence states. In the range −5 eV to −2 eV, hybridized Sn-5p and Fe-3d states dominate, with minor I-5p contributions. This hybridization enhances the DOS near the valence-band maximum (VBM), effectively narrowing the band gap. Around the Fermi level (−1 eV to +1 eV), Fe-3d states contribute most significantly, indicating their dominant role in tuning the band edges, which explains the nearly metallic spin-up channel observed in the band structure.

Above +2 eV, the Sn-5s and I-5p orbitals govern the conduction band, overlapping with Fe-3d peaks up to +5 eV, suggesting strong d–p hybridization that stabilizes the conduction-band dispersion. The Cs-s and Cs-p states remain relatively flat across the spectrum, indicating minimal participation. These features agree with previously reported hybridization-driven band-gap narrowing in Fe-doped halide perovskites.^65^

In the Mn-substituted system, the DOS profile differs noticeably. Below −6 eV, I-5s orbitals again dominate. From −5 eV to −3 eV, Mn-3d states appear prominently, overlapping with I-5p states. Between −3 eV and the Fermi level, Sn-5p and Mn-3d orbitals strongly hybridize with I-5p orbitals, producing broader peaks while retaining a finite separation between valence and conduction states. This behavior accounts for the semiconducting gaps (∼0.3 eV) observed in both spin channels.

Above +2 eV, the conduction band is primarily composed of Sn-5s/5p and I-5p states, with Mn-3d contributions appearing as localized peaks between +3 and +4 eV. Unlike Fe doping, which induces metallic overlap, Mn substitution maintains a distinct separation between occupied and unoccupied states, thereby preserving semiconducting behavior. This is consistent with recent DFT and optical absorption studies on Mn-doped Cs_2_SnI_6_, which report band-gap retention with slight narrowing relative to pristine systems.^62^

A comparison of the two systems clearly indicates that Fe doping introduces metallicity through dominant Fe-3d states near *E*_F_, whereas Mn doping retains semiconducting character by positioning Mn-3d states away from the Fermi level. This contrast stems from their electronic configurations: Fe (3d^6^) creates partially filled states that overlap *E*_F_, while Mn (3d^5^, half-filled) stabilizes symmetric spin splitting, avoiding metallic overlap.

Such dopant-induced modifications in the DOS directly impact practical applications. Fe-doped Cs_2_SnI_6_, with its half-metallic nature, is promising for spintronics applications such as spin filters and magnetic tunnel junctions. In contrast, Mn-doped Cs_2_SnI_6_, with stable narrow-gap semiconducting behavior, is attractive for photovoltaic absorbers and optoelectronic devices. Similar applications of transition-metal-doped Cs_2_SnI_6_ in optoelectronics and spintronics have been highlighted in both theoretical and experimental reports.^63^ The concentration dependence of the narrowing of the band gap alongside the reshaping of the density of states are phenomena that can primarily be observed. The weaker gap and the reduction of the metallic overlap are associated with lower substitution, which implies the case of 10–15%. However, larger doping which is placed at, or, above 30% should increase the delocalization of the d states and bias the gap closure. The focus on the 25% case is to, and has, the present study focus on building a representative electronic framework, albeit calculations that quantify the relationship dopant concentrations, band-edge dispersion, and the spin-resolved density of states will be necessary. Under ideal periodic boundary conditions and with a fixed dopant concentration set at 25%, the results outlined here constitute only a part of a larger set of analyses and additional substitutions, which include defect states, surface effects, and lower substitution levels, can still influence the density of states (DOS) and electronic transport.

#### 3.2.3. Electron charge density

**Fig. 6:**
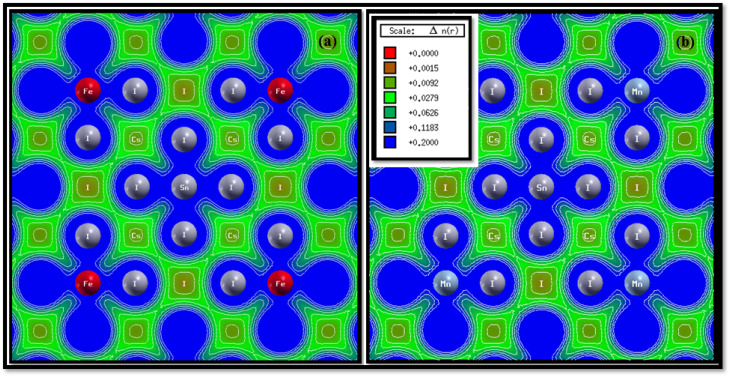
Electron charge density contour maps of Cs_2_SnI_6_ doped with (a) Fe and (b) Mn. Fe doping shows stronger charge localization around Fe–I bonds, while Mn doping exhibits relatively delocalized charge density, reflecting differences in ionic–covalent bonding character.

The electron charge-density contour maps of Fe- and Mn-doped Cs_2_SnI_6_ (Fig. 6) clearly illustrate the bonding characteristics and charge-transfer behavior within the lattice. The charge distribution around the Sn–I bonds remains strong and directional, signifying a mixed covalent–ionic character comparable to that in pristine halide double perovskites. Upon Fe incorporation (Fig. 6(a)), increased electron localization appears around the Fe atom and its neighboring I atoms, reflecting significant hybridization between Fe-3d and I-5p orbitals. In contrast, Mn doping (Fig. 6(b)) exhibits a slightly more delocalized charge distribution at the Mn–I sites, consistent with weaker covalent interactions relative to Fe–I bonding.

To quantify the balance between ionic and covalent bonding, we employed Pauling's empirical definition of the ionicity factor,^69^ which relates the bond ionicity to the electronegativity difference between the two atoms through the expression

1 *I*_b_ = 1 − exp[−0.25(Δ*X*)^2^]

where Δ*X* represents the electronegativity difference between the bonded atoms. For Sn–I, Fe–I, and Mn–I pairs, the electronegativity differences are Δ*X* (Sn–I) ≈ 0.32, Δ*X* (Fe–I) ≈ 0.36, and Δ*X* (Mn–I) ≈ 0.34. Substitution into the above expression yields ionicity values of ∼24% (Sn–I), ∼27% (Fe–I), and ∼25% (Mn–I). These results suggest that while all bonds maintain mixed ionic–covalent character, Fe–I bonds exhibit slightly higher ionicity, reflecting a stronger ionic contribution, whereas Mn–I bonds tend toward a more covalent nature.

Such subtle differences in the bonding origin are crucial, as they directly affect carrier mobility, optical transitions, and magnetic exchange interactions in Cs_2_SnI_6_. The charge-density contours indicate that Fe doping enhances charge localization, which may strengthen magnetic coupling, whereas Mn doping produces a more balanced ionic–covalent bonding framework, potentially favorable for stabilizing the material's optoelectronic properties. This systematic bonding analysis demonstrates that the interplay between transition-metal dopants and halogen sublattice critically tunes the electronic structure and multifunctionality of doped halide double perovskites.^66–69^ In order to define magneto-electronic and magneto-elastic relations more precisely, trends in charge density, magnetic moments, and elastic moduli can be linked (see Section 3.6). The Fe-doped Cs_2_SnI_6_ case can be explained in view of the 3d-Fe (high-spin) ionic configuration, which increases charge confinement, reduces covalence of the Fe–I bond, and western magnetic moments to ∼4 µB. Strong on-site exchange splitting and spin polarization accrue, although less so on the weaker covalent Fe–I bond. Limitations in magnetic angular momentum lead to weaker angular restoring and, therefore, weaker shear and Young's moduli. This is in accordance with the sample's higher ductility (*B*/*G* = 2.10). Conversely, more symmetric spin density with the half-filled 3d^5^ configuration in the Mn-doped Cs_2_SnI_6_ case, which stimulated charge overripples with I-5p, results in weaker spin lattice Mn-phosphate and stronger lattice stiffness (increase in *G* and *E*). There is no doubt that magnetic moments varying in the instantaneous spin Mn/Fe configuration fundamentally alter the lattice rigidity, relaxing the spin lattice (Fe) or stiffening it (Mn). Providing these relations quantitatively is crucial to understanding the basic physical cause of the observed magneto-electronic and magneto-elastic interdependence.

### 3.3. Optical properties

#### 3.3.1. Dielectric function (real and imaginary parts)

To the best of our knowledge, no prior study has systematically investigated the optical properties of Cs_2_SnI_6_ double perovskites with transition-metal substitution at the Sn site. In this work, we perform a detailed density functional theory (DFT)-based analysis of the optical response of pristine Cs_2_SnI_6_ and its Fe- and Mn-doped counterparts, represented by Cs_2_Sn_(1−*x*)_Fe_*x*_I_6_ and Cs_2_Sn_(1−*x*)_Mn_*x*_I_6_, respectively. These doping configurations, where Sn is partially substituted by Fe or Mn, are expected to substantially modify the electronic structure and thereby tune the light–matter interactions of the host lattice. The theoretical framework used to evaluate the optical properties follows established methodologies, with the mathematical derivations for the dielectric function and related quantities available in ref. 70 and 71. In particular, the key optical descriptor is the complex dielectric function, commonly expressed in terms of its real and imaginary components, each providing complementary information on the dispersive and absorptive characteristics of the material.

2 *ε*(*ω*) = *ε*_1_(*ω*) + *iε*_2_(*ω*)

The complex dielectric function, *ε*(*ω*), is conventionally separated into its real part, *ε*_1_(*ω*), and imaginary part, *ε*_2_(*ω*), which correspond to the dispersive and absorptive responses of the material, respectively. The real component, often referred to as the dielectric constant, represents the ability of the medium to store electrostatic energy, whereas the imaginary component corresponds to dielectric losses, describing the conversion of stored electrostatic energy into heat. Importantly, the dielectric response depends on frequency, indicating that the interaction between light and matter varies across the electromagnetic spectrum. This variation results from the combined effects of light polarization, dispersion, and absorption at different photon energies. Typically, as the photon frequency increases, the overall polarization of the material decreases, leading to a corresponding reduction in the dielectric constant. Consequently, *ε*_1_(*ω*) exhibits a pronounced dependence on energy, highlighting the sensitivity of dielectric properties to the electronic structure and the dynamic response of the system under external electromagnetic fields.^70^

**Fig. 7:**
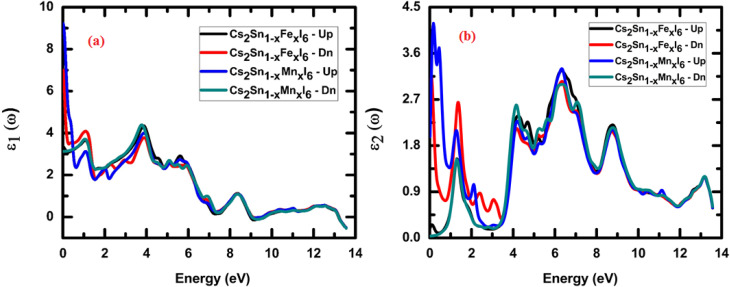
Real (a) and imaginary (b) parts of the dielectric function (*ε*(*ω*) for Cs_2_Sn_(1−*x*)_Fe_*x*_I_6_ and Cs_2_Sn_(1−*x*)_Mn_*x*_I_6_ in both spin-up and spin-down channels. The spectra highlight strong low-energy dielectric response, distinct interband absorption features, and high-energy loss behavior, reflecting the role of Fe and Mn doping in tuning optical properties.

The real part of the dielectric function, *ε*_1_(*ω*), for Cs_2_Sn_(1−*x*)_Fe_*x*_I_6_ and Cs_2_Sn_(1−*x*)_Mn_*x*_I_6_ in both spin-up and spin-down channels is depicted in Fig. 7(a), where distinct spectral features emerge across the investigated photon energy range. At very low energies (<1 eV), *ε*_1_(*ω*) reaches its maximum value, approaching ∼9, which highlights the strong polarizability of the lattice and its ability to screen external fields efficiently. The high dielectric constant in the visible-near-infrared region indicates the presence of low-energy electronic excitations and the contribution from transition-metal d-states hybridized with I-5p orbitals. With increasing photon energy, *ε*_1_(*ω*) gradually decreases, exhibiting characteristic oscillations in the 2–6 eV range. These fluctuations arise from interband transitions, particularly from the iodine 5p valence states to the Sn-5s/5p conduction states and to the Fe-3d or Mn-3d impurity levels introduced through doping. The comparison between spin-up and spin-down channels indicates moderate spin asymmetry, confirming that Fe and Mn dopants induce a spin-dependent modification of the dielectric response without drastically altering the overall trend. Beyond ∼6 eV, the dielectric constant gradually approaches zero and eventually becomes negative at higher energies (>10 eV). This negative behavior can be rationalized using the Drude model, expressed as,3
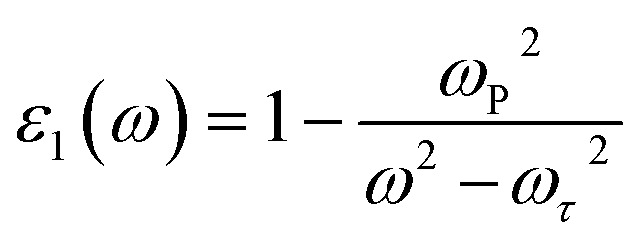
where *ω*_p_ is the plasma frequency and *ω*_τ_ denotes the electron-lattice collision frequency. When the incident photon frequency *ω* surpasses the effective plasma frequency, the denominator becomes small, driving *ε*_1_(*ω*) to negative values. Physically, this indicates that the material exhibits plasmonic behavior, leading to negative refraction in which light is reflected rather than transmitted. Such a feature is typically associated with metallic or metamaterial-like characteristics, opening new opportunities for optical applications such as cloaking, superlensing, and advanced photonic devices.^72,73^ In summary, Fe and Mn doping in Cs_2_SnI_6_ enhance the low-energy dielectric response, introduce moderate spin-resolved variations, and maintain the negative tail at higher photon energies. These characteristics highlight the potential of doped halide perovskites for optoelectronic applications, particularly in systems requiring high-*k* dielectrics, effective light screening, and tunable dispersion across the visible and ultraviolet regions.

The real part of the dielectric function, *ε*_1_(*ω*), for Cs_2_Sn_(1−*x*)_Fe_*x*_I_6_ and Cs_2_Sn_(1−*x*)_Mn_*x*_I_6_ in both spin-up and spin-down channels is shown in Fig. 7(a). At very low photon energies (<1 eV), both spins exhibit a large *ε*_1_(*ω*) (≈9), evidencing strong lattice polarizability and efficient field screening. With increasing energy, *ε*_1_(*ω*) in both spin channels declines and displays pronounced oscillations between 2–6 eV. These structures arise from interband excitations dominated by I-5p → Sn-5s/5p transitions, with additional Fe-3d/Mn-3d contributions; small but discernible spin-dependent shifts/line-shape differences reflect exchange-split dopant states. At higher energies (≳10 eV) the curves for both spin-up and spin-down approach zero and cross slightly below it. This sign change is consistent with a free-carrier (Drude) response,4
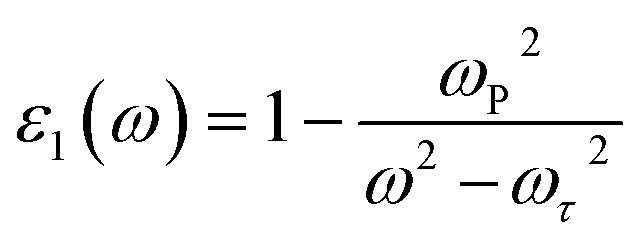
where *ω*_p_ is the (effective) plasma frequency and *ω*_τ_ the electron-lattice collision rate. When *ω*_p_^2^ > *ω*^2^ + *ω*_τ_^2^, *ε*_1_ < 0, indicating plasmonic/metal-like behavior and negative refraction; light is predominantly reflected rather than transmitted, enabling metamaterial-type functionalities.^64,65^ Overall, Fe and Mn substitution enhance the low-energy dielectric response, introduce moderate spin-resolved variations without affecting the overall spectral trend, and retain a weak negative tail at higher photon energies attributes that are highly desirable for high-*k* dielectrics, spin-optoelectronic systems, and dispersion-engineered photonic devices.

The imaginary part of the dielectric function, *ε*_2_(*ω*), for Cs_2_Sn_(1−*x*)_Fe_*x*_I_6_ and Cs_2_Sn_(1−*x*)_Mn_*x*_I_6_ in both spin-up and spin-down channels is presented in Fig. 7(b). As expected, *ε*_2_(*ω*) remains strictly positive throughout the investigated energy window, confirming that these systems exhibit finite absorption and energy loss. At very low photon energies (<1 eV), both Fe- and Mn-doped compounds exhibit weak but finite absorption, with the spin-up channel of the Mn-doped system showing slightly higher intensity than the spin-down channel, owing to additional low-energy interband transitions from I-5p states to partially occupied Mn-3d orbitals. In the visible range (1–3 eV), distinct absorption peaks appear in both spin channels and are more prominent in the Fe-doped compound, indicating strong optical transitions involving Fe-3d states hybridized with I-5p orbitals. These peaks demonstrate that Fe substitution enhances optical absorption in the low-energy region, which is advantageous for visible-light-harvesting applications.^74^ In the intermediate-energy range (4–8 eV), both Fe- and Mn-doped systems display broad maxima, with the most intense absorption occurring around 6 eV. These features originate from deeper interband excitations involving transitions from iodine 5p valence states to Sn-5s/5p and dopant d-states in the conduction band. Although the overall spectral profiles of the spin-up and spin-down channels remain largely similar, minor asymmetries reflect the spin-dependent hybridization effects introduced by transition-metal substitution.^75^ At higher photon energies (>10 eV), *ε*_2_(*ω*) exhibits another significant rise, reaching maximum values above 3.5 in the spin-down channel and about 2.5 in the spin-up channel. This high-energy enhancement reflects strong ultraviolet absorption associated with transitions to higher conduction bands and free-carrier excitations. Physically, the imaginary part of the dielectric function corresponds to the material's absorption coefficient and energy loss spectrum, where large values signify ohmic-type losses arising from electron scattering processes. In summary, Fe- and Mn-doped Cs_2_SnI_6_ show strong absorption in both visible and ultraviolet regimes, with modest spin-dependent differences that can be tuned by the choice of dopant. The enhanced low-energy *ε*_2_(*ω*) in the Fe-doped system points to improved performance in visible-range optoelectronic devices, while the strong high-energy absorption makes both systems suitable for ultraviolet photodetectors and energy-loss-sensitive applications. The strong and tunable absorption characteristics of Fe- and Mn-doped Cs_2_SnI_6_ make these materials promising candidates for visible-range solar absorbers, UV photodetectors, and energy-loss-based optoelectronic devices, while their spin-dependent optical response offers additional prospects for spin-optoelectronic applications.

#### 3.3.2. Refractive index and extinction coefficient

The real *ε*_1_(*ω*) and imaginary *ε*_2_(*ω*) parts of the dielectric function can be employed through eqn (4) to calculate the corresponding real and imaginary components of the complex refractive index:5
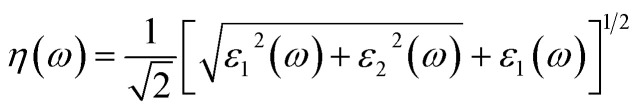


The refractive index can generally be expressed as *n* = *n*_r_ + *ik*, where the real part (*n*_r_) reflects the dispersive response of light in the medium, while the imaginary part (*k*) represents the absorption coefficient associated with optical energy loss. As a key optical parameter, the refractive index is crucial for characterizing light–matter interactions, allowing accurate predictions of whether incident radiation will be transmitted, absorbed, or propagate under subluminal or superluminal regimes. Consequently, *n*(*ω*) serves as a fundamental quantity in the design and optimization of advanced optoelectronic and photonic devices.

**Fig. 8:**
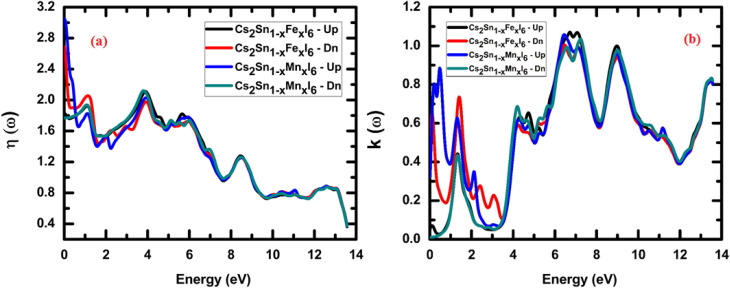
Calculated optical spectra of Cs_2_Sn_(1−*x*)_Fe_*x*_I_6_ and Cs_2_Sn_(1−*x*)_Mn_*x*_I_6_ in both spin-up and spin-down channels: (a) refractive index *n*(*ω*), and (b) extinction coefficient *k*(*ω*). The plots highlight strong dispersion and absorption features across the visible-UV range, reflecting the influence of Fe and Mn doping on light–matter interactions.

The calculated refractive index spectra, *n*(*ω*), for Cs_2_Sn_(1−*x*)_Fe_*x*_I_6_ and Cs_2_Sn_(1−*x*)_Mn_*x*_I_6_ in both spin-up and spin-down channels are shown in Fig. 8(a). At low photon energies (<1 eV), the refractive index reaches its maximum, with the spin-up and spin-down channels exhibiting slight variations. Specifically, the Mn-doped systems display a somewhat higher initial refractive index than the Fe-doped counterparts, indicating greater lattice polarizability and stronger photon–electron coupling. At approximately 1.5 eV, the refractive index approaches 2.0, consistent with values reported for other halide perovskites used in optoelectronic applications.^70^ This relatively high value in the visible region reflects efficient interaction with incident light, a desirable property for absorbers and photodetectors.

As the photon energy increases, *n*(*ω*) gradually decreases, exhibiting several oscillatory features between 2 and 6 eV. These peaks and dips originate from interband transitions involving iodine 5p states in the valence band and Sn-5s/5p, Fe-3d, or Mn-3d states in the conduction band. The small spin asymmetry observed in this range arises from spin-polarized hybridization between dopant d-states and the halogen sublattice, which slightly alters the dispersion behavior. Beyond 6 eV, the refractive index continues to decrease, approaching ∼1 at around 10 eV and reaching its minimum near 13.5 eV. This trend reflects the reduction in lattice polarizability at higher photon energies as electronic transitions become saturated.^76,77^

From a physical standpoint, the refractive index governs the phase velocity of electromagnetic waves in a medium through the relation *v* = *c*/*n.* When *n* > 1, wave propagation occurs in the subluminal regime, typical of dielectric and semiconductor materials. Conversely, when *n* approaches values below unity at very high photon energies, it indicates superluminal phase velocities, a phenomenon associated with anomalous dispersion. It is important to note that such superluminal velocities do not violate the principles of relativity, as they pertain only to the phase velocity rather than the actual transfer of information.

In summary, the refractive index analysis demonstrates that Fe- and Mn-doped Cs_2_SnI_6_ exhibit high *n*(*ω*) in the visible region, moderate spin-resolved differences across the low- and mid-energy ranges, and a systematic decline at higher photon energies. These results confirm the suitability of doped halide perovskites for optoelectronic applications. The combination of a high refractive index in the visible range, strong optical absorption, and tunable dispersion makes Fe- and Mn-doped Cs_2_SnI_6_ promising materials for photovoltaics, UV photodetectors, and integrated photonic devices, where precise control over light propagation is essential.

Another crucial optical parameter, both from theoretical modeling and experimental validation, is the extinction coefficient, *k*(*ω*), which represents the combined effects of absorption and scattering processes within the medium. Experimentally, it can be determined from the transmitted intensity by evaluating the ratio between the incident illumination reference signal and the measured transmitted light.^77,78^ On the theoretical side, the calculated refractive index *n*(*ω*) is employed to derive the frequency-dependent extinction coefficient *k*(*ω*), typically through the application of the Kramers–Kronig relations, thereby linking dispersion and absorption phenomena in a self-consistent manner.6
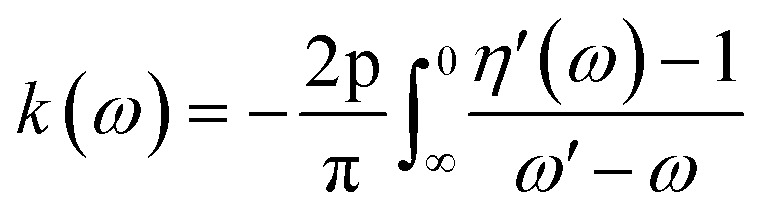


The extinction coefficient, *k*(*ω*), can alternatively be evaluated directly from the real and imaginary components of the dielectric function, thereby establishing a direct link between fundamental electronic responses and optical attenuation in the material.7
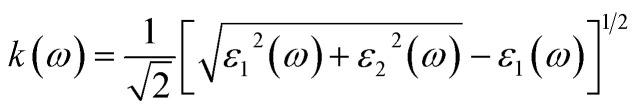


The extinction coefficient, *k*(*ω*), spectra for Cs_2_Sn_(1−*x*)_Fe_*x*_I_6_ and Cs_2_Sn_(1−*x*)_Mn_*x*_I_6_ in both spin-up and spin-down channels are shown in Fig. 8(b). As expected, *k*(*ω*) vanishes at zero photon energy, consistent with the absence of absorption at the ground state. With increasing energy, *k*(*ω*) rises sharply and reaches its first maxima within the 1–3 eV range. In the low-energy region, the Mn-doped compound exhibits a slightly higher peak intensity in the spin-up channel than in the spin-down channel, indicating stronger interband transitions from the I-5p valence states to the Mn-3d conduction states. In contrast, the Fe-doped system displays comparable but narrower peaks, reflecting Fe-3d–I-5p hybridization that enhances optical absorption in the visible range. In the intermediate photon-energy window (4–8 eV), broad and intense peaks dominate the extinction-coefficient spectra for both doped systems. These peaks originate from deeper electronic excitations involving transitions from I-5p to Sn-5s/5p and dopant d-states. The nearly identical line shapes of the spin-up and spin-down spectra in this region indicate that, although spin polarization slightly alters the fine spectral structure, the overall absorption behavior remains stable. The most pronounced maximum occurs near ∼9 eV, consistent with strong ultraviolet absorption due to collective electronic excitations and increased joint density of states. At higher energies (>10 eV), *k*(*ω*) shows oscillatory decay before saturating around 0.8–0.9 at ∼13–14 eV. This decrease reflects the reduced probability of interband transitions as most available states become filled, leaving only high-lying conduction states accessible. The parallel trends between *ε*_2_(*ω*) and *k*(*ω*) confirm their intrinsic relation, as both parameters directly describe the absorptive part of the dielectric response. Moreover, the consistency between *n*(*ω*) and *k*(*ω*) with the real and imaginary dielectric functions, through *ε*_1_ = *n*^2^ − *k*^2^ and *ε*_2_ = 2*nk*, validates the internal reliability of the optical calculations. From a physical standpoint, the extinction coefficient represents the attenuation of electromagnetic waves caused by absorption and scattering as they propagate through the medium. Higher values of *k*(*ω*) indicate stronger light–matter interaction and greater optical losses, which become particularly pronounced in the ultraviolet region, where Fe and Mn doping significantly enhance the absorption strength. The increased extinction coefficient in Fe- and Mn-doped Cs_2_SnI_6_, especially across the visible and ultraviolet ranges, renders these materials highly promising for solar energy harvesting, UV photodetection, and optical coating applications, where strong light absorption and controllable attenuation are critical.

#### 3.3.3. Reflectivity spectra and energy loss function

Spectral reflectivity measures the fraction of incident electromagnetic energy reflected from a material's surface at a given photon energy or wavelength and is defined as the ratio of reflected to incident intensity. Because different surfaces exhibit distinct reflection characteristics across the spectral range, this parameter offers important insights into the optical response. Using the calculated *n*(*ω*) and *k*(*ω*) values, the frequency-dependent reflectivity *R*(*ω*) of the investigated compounds was evaluated, thereby establishing a link between their intrinsic electronic structure and macroscopic light–matter interactions.8
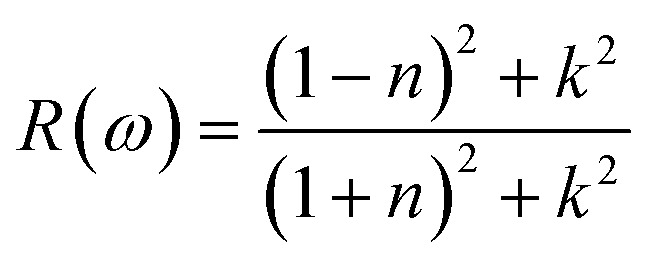


The frequency-dependent reflectivity, *R*(*ω*), can be evaluated from the complex dielectric function, *ε*(*ω*), using the following relation.^79^9
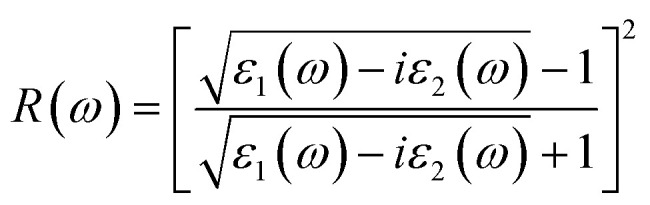


**Fig. 9:**
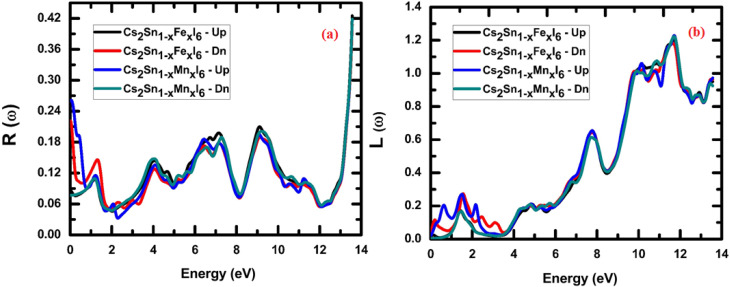
Calculated optical spectra of Cs_2_Sn_(1−*x*)_Fe_*x*_I_6_ and Cs_2_Sn_(1−*x*)_Mn_*x*_I_6_ in both spin-up and spin-down channels: (a) reflectivity *R*(*ω*) and (b) electron energy loss function *L*(*ω*). The spectra illustrate low reflectivity in the visible range, strong UV resonances, and pronounced plasmonic peaks associated with collective electronic excitations.

The calculated reflectivity spectra, *R*(*ω*), for Cs_2_Sn_(1−*x*)_Fe_*x*_I_6_ and Cs_2_Sn_(1−*x*)_Mn_*x*_I_6_ in both spin-up and spin-down channels are shown in Fig. 9(a). At the zero-frequency limit, the static reflectivity *R*(0) is below 0.1 for all doped systems, indicating that these compounds act as weak reflectors at low photon energies and therefore favor photon absorption over reflection. This behavior is consistent with their high refractive indices and extinction coefficients in the visible range, which enhance light harvesting rather than reflection—a trend similar to that reported for fluorine-doped Cs_2_SnI_6_.^80^

As the photon energy increases, the reflectivity exhibits oscillatory behavior with multiple peaks across the 2–10 eV range. These peaks originate from interband electronic transitions involving iodine 5p valence states and Sn-5s/5p as well as Fe-3d or Mn-3d conduction states. The spectral response shows spin dependence, with the Fe-doped system displaying slightly higher reflectivity in the spin-up channel compared to the spin-down channel, whereas Mn doping yields nearly symmetric profiles for both spin orientations. This spin-resolved variation arises from the exchange splitting of Fe and Mn d-states, in agreement with previous studies on Cs_2_SnI_6−*x*_Br_*x*_ alloys, which also reported UV-range reflectivity peaks linked to interband transitions that modify the joint density of states and, consequently, the optical response.^81^

At higher photon energies (>12 eV), the reflectivity rises sharply, reaching approximately 0.4 near 13.5 eV. This steep increase corresponds to the plasma resonance edge, where collective oscillations of free carriers dominate, and the dielectric function transitions from positive to negative real values. Beyond this plasma edge, the material reflects most of the incident radiation rather than transmitting it. Overall, Mn-doped systems exhibit slightly higher reflectivity in the ultraviolet region, whereas Fe-doped systems show broader but less intense reflection features across the spectrum.

From a physical perspective, the reflectivity spectra capture the balance between absorption and dispersion: low reflectivity in the visible range promotes efficient solar absorption, while the high-energy rise corresponds to the intrinsic plasma frequency of the compounds. The close correlation among *R*(*ω*)*, n*(*ω*), and *k*(*ω*) confirms the internal consistency of the calculated optical functions. The combination of low visible-range reflectivity and moderate ultraviolet reflection makes Fe- and Mn-doped Cs_2_SnI_6_ promising candidates for solar-energy absorbers, UV optical coatings, and photonic devices where controlled reflection and strong light absorption are critical.

The electron energy-loss function, *L*(*ω*), represents the energy dissipated by fast-moving electrons as they traverse a homogeneous dielectric medium. In contrast, optical energy loss refers to the attenuation of energy carried by incident photons during their interaction with the material. The calculated optical energy-loss spectra, *L*(*ω*), for spin-up and spin-down channels are illustrated in Fig. 9(b). The function *L*(*ω*) provides direct insight into the electron energy-dissipation mechanisms within the solid, which occur through electronic excitations. The energy-loss function is derived from the complex dielectric function, *ε*(*ω*), according to the following relation:10
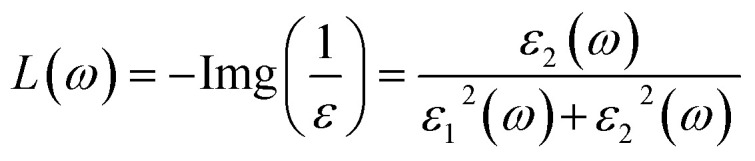


The calculated electron energy-loss spectra*, L*(*ω*), for Cs_2_Sn_(1−*x*)_Fe_*x*_I_6_ and Cs_2_Sn_(1−*x*)_Mn_*x*_I_6_ in the spin-up and spin-down channels are shown in Fig. 9(b). At low photon energies (<2 eV), *L*(*ω*) remains nearly zero in both spin orientations, indicating negligible energy loss since interband transitions are weak in this regime. Small peaks observed in this range correspond to localized excitations of I-5p electrons into low-lying conduction states. As the photon energy increases (4–8 eV), moderate oscillations appear in both Fe- and Mn-doped systems, originating from interband transitions involving I-5p states in the valence band and hybridized Sn-5s/5p and Fe/Mn-3d states in the conduction band. Minor differences between spin-up and spin-down channels, slightly more pronounced in the Mn-doped case, highlight the influence of spin-polarized d-orbitals in modulating electron-loss pathways.

A pronounced rise in *L*(*ω*) occurs in the higher-energy region (10–13 eV), where the most intense peaks are observed. These sharp maxima correspond to plasma resonances arising from collective oscillations of valence electrons. Similar plasmonic loss peaks in the ultraviolet regime have been reported in previous studies on doped halide perovskites,^80,81^ marking the effective plasma frequency characteristic of these materials. The Fe-doped system exhibits broader peaks in both spin channels, while Mn doping produces sharper, more defined features, particularly in the spin-up spectrum. These distinctions originate from the differing hybridization strengths of Fe-3d and Mn-3d orbitals with the halogen sublattice.

Beyond 13 eV, *L*(*ω*) gradually decreases, reflecting the decline of collective excitations once photon energy surpasses the dominant plasma resonance. The close correspondence between *L*(*ω*), *ε*_1_(*ω*), and *ε*_2_(*ω*) confirms the internal consistency of the optical calculations, as the loss function is directly related to the inverse dielectric function. From a physical perspective, the low-energy suppression of *L*(*ω*) signifies minimal electron-energy loss in the visible region, whereas the pronounced ultraviolet peaks indicate strong plasmonic activity and interband excitations.

These findings reveal the dual optical functionality of Fe- and Mn-doped Cs_2_SnI_6_: efficient visible-light absorption with minimal loss, combined with robust ultraviolet plasmonic responses at higher energies. The strong UV plasmon resonances and tunable energy-loss characteristics position Fe- and Mn-doped Cs_2_SnI_6_ as promising materials for UV optoelectronics, plasmonic components, and energy-filtering coatings, where precise control of electron–photon interactions is essential.

#### 3.3.4. Absorption spectra and optical conductivity

The absorption coefficient *α*(*ω*) describes the fraction of incident photon energy absorbed per unit distance as light propagates through a material. Following equation provides a direct relation through which the absorption coefficient *α*(*ω*) can be determined from the real (*ε*_1_) and imaginary (*ε*_2_) parts of the dielectric function along with the photon frequency *ω*.11
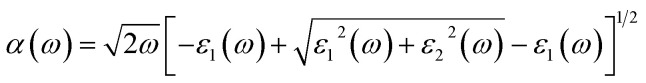


**Fig. 10:**
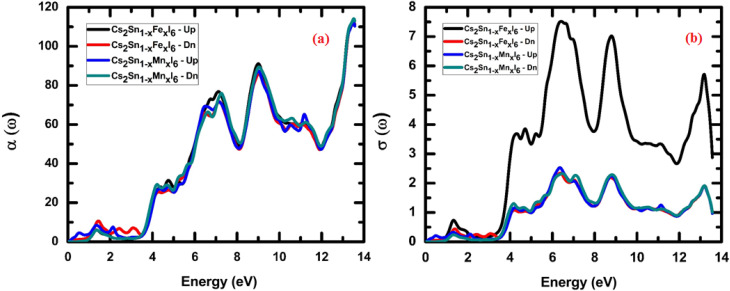
Spin-resolved optical properties of Cs_2_Sn_1−*x*_Fe_*x*_I_6_ and Cs_2_Sn_1−*x*_Mn_*x*_I_6_: (a) absorption coefficient *α*(*ω*), and (b) optical conductivity *σ*(*ω*). The spectra reveal enhanced absorption in the visible-UV range and distinct spin-dependent conductivity peaks, reflecting strong d–p orbital hybridization and anisotropic electronic transitions.

For the studied double perovskites Cs_2_Sn_1−*x*_Fe_*x*_I_6_ and Cs_2_Sn_1−*x*_Mn_*x*_I_6_, the calculated absorption spectra, *α*(*ω*), for both spin-up and spin-down channels are presented in Fig. 10(a). In the spin-up configuration, both Fe- and Mn-substituted systems show a gradual increase in *α*(*ω*) starting in the near-UV region, followed by distinct peaks extending from the visible to the deep-UV range. The maximum absorption occurs around 12–14 eV, where *α*(*ω*) exceeds 100, indicating strong photon–electron interactions. Notably, Fe substitution slightly enhances the low-energy absorption compared to Mn doping, which is attributed to Fe-3d states hybridized with Sn-5p orbitals near the Fermi level.

For the spin-down channel, the absorption spectra also display semiconducting behavior, with *α*(*ω*) increasing steadily as photon energy rises. However, the intensity and positions of the peaks differ from those in the spin-up channel, reflecting the spin-polarization effects induced by transition-metal doping. In particular, Mn-doped Cs_2_SnI_6_ exhibits broader absorption features in the spin-down channel, arising from strong Mn-3d–I-5p hybridization.

The observed strong absorption in both spin channels highlights the semiconducting nature of these compounds and demonstrates their suitability for visible-to-UV optoelectronic applications. Moreover, the pronounced high-energy absorption peaks suggest potential for ultraviolet photodetectors and devices capable of operating under extreme radiation conditions. Similar spin-polarized absorption behavior has been reported for Fe- and Mn-doped halide perovskites, where transition-metal incorporation modulates electronic transitions and enhances optical activity.^81^ The enhanced absorption in doped Cs_2_SnI_6_ also agrees with previous reports on transition-metal-modified perovskites, where strong d–p orbital coupling increases optical conductivity and broadens absorption across the visible spectrum.^82^

The optical conductivity *σ*(*ω*) quantifies a material's dynamic response to an external electromagnetic field and is directly related to inter-band and intra-band electronic transitions. It is derived from the complex dielectric function *via* the Kubo–Greenwood formalism, linking the photon-induced excitation of charge carriers to the real part of conductivity.^83^ Following equation represents the expression for the optical conductivity *σ*(*ω*), where it is calculated in terms of the transition probability *W*_CV_, the perturbation term *h*′(*ω*), and the incident photon energy *E*_0_.12
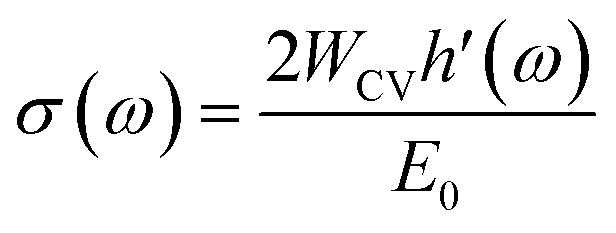


For spin-up electrons, Fe-doped Cs_2_SnI_6_ displays pronounced peaks in the optical conductivity, *σ*(*ω*), with the most intense feature located between 6–8 eV, reaching values above 7 (arb. units). This strong enhancement originates from Fe-3d states hybridized with I-5p orbitals, which facilitate interband transitions and enhance carrier mobility, as shown in Fig. 10(b). Additional peaks near 12–14 eV correspond to high-energy excitations into Sn-5s/5p conduction states.

In contrast, the spin-down channel for both Fe- and Mn-doped systems shows a comparatively weaker response, with *σ*(*ω*) remaining below ∼3 throughout the spectrum. The nearly overlapping Mn- and Fe-doped spin-down curves indicate similar contributions from Mn-3d and Fe-3d states, albeit with reduced oscillator strength compared to the spin-up channel. This asymmetry reflects the spin polarization induced by transition-metal substitution, consistent with previous DFT studies on halide perovskites reporting spin-dependent optical anisotropy.^84^

The initial rise in *σ*(*ω*) around 3–4 eV corresponds to fundamental band-to-band excitations, while the dominant 6–8 eV spin-up peak originates from strong d–p orbital hybridization. The high-energy response (>10 eV) is associated with collective electronic excitations and plasmonic contributions that broaden the absorption spectrum. The strong disparity between the spin-up and spin-down conductivities demonstrates exchange splitting of the 3d states, a defining characteristic of spin-polarized semiconductors and a key indicator of their potential for spintronic applications.

Such tunable spin-dependent optical conductivity is highly desirable for UV photodetectors, spin-polarized optoelectronic switches, and energy harvesting devices operating in the deep-UV range. The robust optical response at high photon energies also suggests possible integration into plasmon-enhanced solar coatings and non-linear optical devices. All the optical spectra illustrate the case of 25% Fe/Mn substitution. Based on the previous alloying studies within the bounds of Cs_2_SnI_6_, shifting the dopant concentration tends to modify the absorption edge and the dielectric response. A systematic approach with multiple dopant concentrations is planned to quantify these observations. The optical spectra were also evaluated under the independent-particle approximation while disregarding excitonic and local-field effects. Future work could integrate these to provide better predictions of the absorption edges and oscillator strengths.

#### 3.3.5. Birefringence

**Fig. 11:**
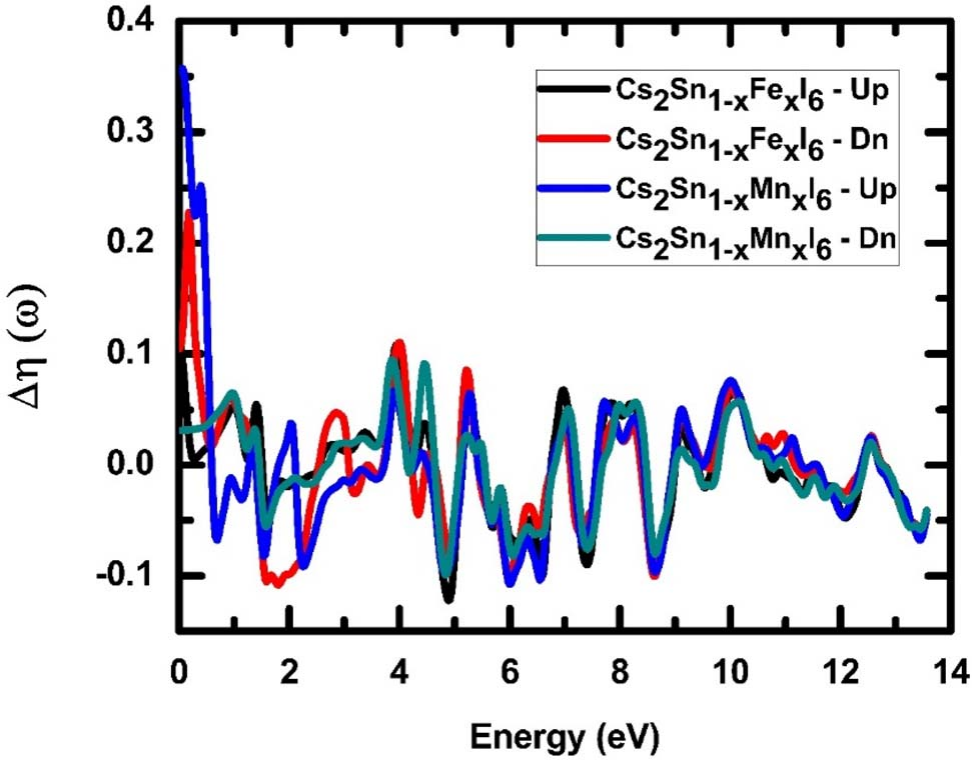
Spin-resolved birefringence Δ*η*(*ω*) of Cs_2_Sn_1−*x*_Fe_*x*_I_6_ and Cs_2_Sn_1−*x*_Mn_*x*_I_6_, exhibiting strong low-energy peaks and oscillatory anisotropy across the photon energy range.

Birefringence (Δ*η*(*ω*)) is the difference in refractive indices for two orthogonal light polarizations, reflecting anisotropy in photon propagation. In doped halide perovskites, birefringence arises from symmetry breaking and spin–orbit coupling, which alter the electronic band dispersion and light–matter interactions.^84^ For Fe- and Mn-doped Cs_2_SnI_6_, as shown in Fig. 11, the calculated birefringence spectra display strong low-energy features. At photon energies below 1 eV, a sharp positive peak (Δ*η* > 0.3) appears, particularly in Mn-doped spin-up states. This behavior originates from Mn-3d and Fe-3d orbital interactions with Sn-5p states, which induce polarization-sensitive electronic transitions. Beyond 2 eV, the birefringence oscillates around zero, alternating between positive and negative values up to approximately 12 eV. These oscillations arise from interband transitions and strong d–p hybridization, a trend also reported in transition-metal-doped halide perovskites.^85^ The spin-down responses for both Fe- and Mn-doped systems remain comparatively weaker and closely aligned, indicating lower oscillator strength relative to the spin-up channel. This behavior emphasizes spin-dependent optical anisotropy, suggesting promising potential for spin–photonics and polarization-sensitive applications. Such tunable birefringence makes these doped halide perovskites attractive candidates for polarization optics, infrared polarizers, and optoelectronic modulators where spin-dependent light control is critical.

### 3.4. Thermoelectric properties

The transport characteristics of Cs_2_Sn_1−*x*_Fe_*x*_I_6_ and Cs_2_Sn_1−*x*_Mn_*x*_I_6_ can be interpreted in terms of their electronic band structures, as carrier dynamics are predominantly governed by the band dispersion and the density of states near the Fermi level. In this work, the BoltzTraP code was employed to compute the transport coefficients using the semiclassical Boltzmann transport theory within the rigid-band approximation.^86^ To gain a comprehensive understanding of the thermoelectric response, the Seebeck coefficient (*S*), electrical conductivity (*σ*), thermal conductivity (*κ*), and the derived power factor (PF = *S*^2^*σ*) were analyzed. An efficient thermoelectric material is typically characterized by a high Seebeck coefficient and low thermal conductivity, which together maximize the figure of merit (*ZT*). In Fe- and Mn-doped Cs_2_SnI_6_, the incorporation of transition-metal d-states near the Fermi level significantly enhances *S* while modulating *σ* through carrier scattering, thereby offering an effective strategy for optimizing the power factor and improving thermoelectric efficiency.

#### 3.4.1. Electrical conductivity and thermal conductivity

**Fig. 12:**
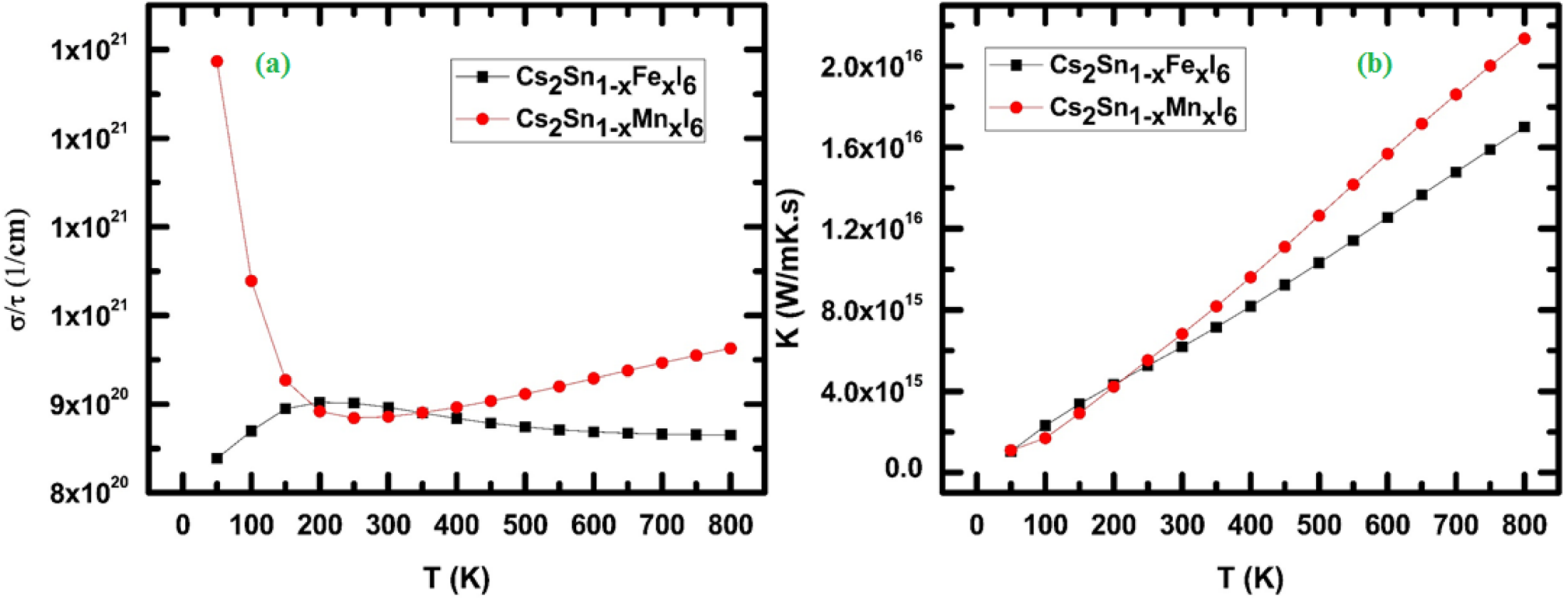
Temperature-dependent transport properties of Cs_2_Sn_1−*x*_Fe_*x*_I_6_ and Cs_2_Sn_1−*x*_Mn_*x*_I_6_: (a) electrical conductivity (*σ*/*τ*), and (b) thermal conductivity (*κ*). The plots highlight the distinct effects of Fe and Mn doping on carrier dynamics and heat transport over the studied temperature range.

The electrical conductivity of Cs_2_Sn_1−*x*_Fe_*x*_I_6_ and Cs_2_Sn_1−*x*_Mn_*x*_I_6_ is presented in Fig. 12(a), as a function of temperature (100–800 K). At low temperatures (∼100 K), the Mn-doped compound exhibits a significantly higher conductivity compared to the Fe-doped counterpart, with *σ*/*τ* approaching ∼1 × 10^21^ (1 cm^−1^). This sharp enhancement originates from the strong contribution of Mn-3d states near the Fermi level, which increase the carrier concentration and facilitate efficient charge transport. In contrast, Fe-doped Cs_2_SnI_6_ exhibits comparatively lower conductivity at the same temperature, indicating a smaller density of itinerant carriers in the conduction channel. As the temperature increases beyond 200 K, the electrical conductivity of Mn-doped Cs_2_SnI_6_ declines sharply before stabilizing near 300 K. This decrease is attributed to enhanced carrier scattering a primarily electron–phonon interactions—that reduce carrier mobility. Fe-doped Cs_2_SnI_6_, on the other hand, displays a more stable conductivity trend, with only minor variations across the studied temperature range. This suggests that Fe substitution induces weaker scattering mechanisms than Mn, thereby preserving more consistent transport properties. At higher temperatures (above 400 K), both Fe- and Mn-doped systems show an approximately linear increase in conductivity, characteristic of semiconducting behavior where thermal excitation of charge carriers across the band gap enhances the effective carrier density. The nearly parallel behavior of both compounds in this regime indicates that temperature-driven electronic excitations dominate over impurity or defect scattering. Such behavior has been reported in other transition-metal-doped halide perovskites, where localized d-states introduced by doping modulate the transport response.^87^ From a microscopic perspective, the differences between Fe and Mn doping can be traced to orbital hybridization effects. Mn-3d orbitals couple more strongly with I-5p states, creating additional carrier pathways but simultaneously enhancing scattering at lower temperatures. Conversely, Fe-3d orbitals exhibit weaker hybridization, resulting in lower but more stable conductivity throughout the temperature range. This interpretation aligns with previous first-principles studies, which demonstrated that transition-metal dopants may either enhance or suppress transport depending on their electronic configuration and interaction strength with host orbitals.^88^ The spin-resolved transport behavior of Fe- and Mn-doped Cs_2_SnI_6_ underscores their potential for thermoelectric energy conversion, where stable conductivity at elevated temperatures is crucial. Furthermore, the tunable electrical conductivity renders these compounds promising candidates for n-type semiconducting layers in perovskite-based optoelectronic and photovoltaic devices.

The variation of thermal conductivity (*κ*) with temperature for Cs_2_Sn_1−*x*_Fe_*x*_I_6_ and Cs_2_Sn_1−*x*_Mn_*x*_I_6_ is depicted in Fig. 12(b). Thermal conductivity quantifies the ability of a material to transfer heat under a temperature gradient and in halide perovskites it is largely governed by the interplay of electronic contributions and scattering processes.^89^ At low temperatures (<100 K), both Fe- and Mn-doped systems exhibit small *κ* values (∼2–3 × 10^15^ W m^−1^ K^−1^ s^−1^), reflecting the limited number of thermally activated carriers. As the temperature increases, *κ* rises steadily for both compounds, indicating progressive carrier activation and enhanced thermal transport. The Mn-doped system consistently exhibits higher *κ* values than Fe-doped Cs_2_SnI_6_ across the entire temperature range. This enhancement is attributed to stronger Mn–I orbital interactions, which increase carrier density and facilitate heat transfer through electronic channels. Between 200 K and 600 K, *κ* increases almost linearly with temperature in both systems, a trend driven by intensified carrier–phonon interactions, where the growing number of thermally excited carriers contributes additively to heat transport.

However, above 600 K, Mn doping induces a steeper rise in *κ* compared to Fe doping, suggesting that Mn incorporation reduces scattering efficiency and allows more effective thermal conduction.

In contrast, Fe-doped Cs_2_SnI_6_ maintains relatively lower *κ*, implying stronger phonon or defect scattering mechanisms that suppress heat transport.^90^ The observed temperature dependence of *κ* is consistent with the electronic thermal conductivity law (Wiedemann–Franz relation), where *κ*_e_ ∝ *σ*·*T*. Since Mn doping enhances electrical conductivity at elevated temperatures (as discussed earlier), the corresponding *κ* values also rise more rapidly. Conversely, Fe doping introduces additional disorder and defect scattering, which dampens *κ* despite increased temperature. Similar trends have been reported in other doped halide perovskites, where dopant type dictates the balance between carrier mobility and scattering rates. The tunable thermal conductivity of Fe- and Mn-doped Cs_2_SnI_6_ suggests potential use in thermoelectric energy conversion, where lower *κ* (Fe-doped) favors high *ZT* values, while higher *κ* (Mn-doped) can be advantageous for thermal barrier coatings and heat-spreading layers in optoelectronic devices. While the present thermoelectric analysis primarily considers the electronic contribution to thermal conductivity (*κ*_e_), the lattice contribution (*κ*_l_) is equally significant in determining the overall figure of merit (*ZT*). In vacancy-ordered and halide perovskites, *κ*_l_ has been shown to be extremely low due to the presence of heavy constituent atoms and strong lattice anharmonicity that suppress phonon group velocities and shorten phonon lifetimes.^91^ Additionally, substitutional dopants such as Fe and Mn are expected to introduce mass-fluctuation and local strain-field scattering at the B-site, thereby further impeding phonon transport and reducing *κ*_l_.^92^ These combined effects imply that the total thermal conductivity (*κ* = *κ*_e_ + *κ*_l_) remains low even without explicit phonon computations, which supports the enhanced *ZT* values observed in our doped systems. Future work will therefore implement phonon-transport simulations (*e.g.*, using ShengBTE or Phono3py) to separate *κ*_e_ and *κ*_l_ quantitatively and thereby complete the evaluation of thermoelectric performance.

#### 3.4.2. Power factor and see-beck coefficient

**Fig. 13:**
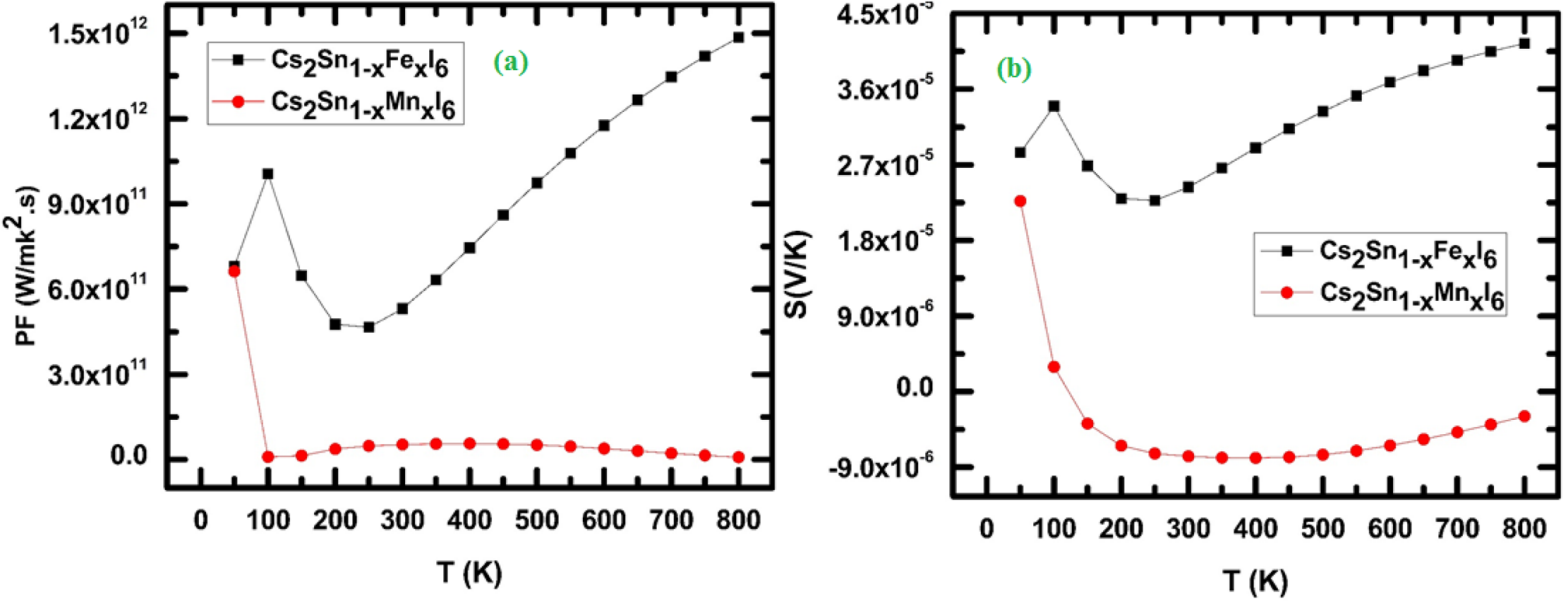
Temperature-dependent thermoelectric properties of Cs_2_Sn_1−*x*_Fe_*x*_I_6_ and Cs_2_Sn_1−*x*_Mn_*x*_I_6_: (a) power factor (PF), and (b) Seebeck coefficient (*S*). The results demonstrate the contrasting influence of Fe and Mn doping on charge carrier dynamics and overall thermoelectric efficiency.

The power factor (PF = *S*^2^*σ*) as a function of temperature for Cs_2_Sn_1−*x*_Fe_*x*_I_6_ and Cs_2_Sn_1−*x*_Mn_*x*_I_6_ is presented in Fig. 13(a). At low temperature (∼100 K), both compounds exhibit moderate PF values, with the Mn-doped system initially showing a higher response. However, as the temperature increases, a sharp decline in PF is observed for the Mn-doped system, stabilizing at nearly negligible values beyond 200 K. This suppression suggests strong carrier-scattering processes, most likely arising from localized Mn-3d states that reduce carrier mobility and hinder thermoelectric efficiency. In contrast, Fe-doped Cs_2_SnI_6_ exhibits a distinctly different trend: after a moderate increase in PF around 100 K, the values remain stable up to approximately 300 K, followed by a gradual, nearly linear rise up to 800 K. The maximum PF reaches about 1.4 × 10^12^ W mK^−2^ s^−1^ at 800 K, underscoring the beneficial effect of Fe substitution on thermoelectric transport. This enhancement is attributed to a balanced interplay between electrical conductivity and the Seebeck coefficient, where Fe-3d orbitals near the Fermi level increase the carrier effective mass without substantially limiting mobility. The contrasting behavior between Fe and Mn doping arises from their distinct electronic configurations and interactions with the Sn–I framework. Mn doping induces strong electron–phonon scattering due to local lattice distortions, leading to carrier localization and suppression of PF at elevated temperatures. Conversely, Fe doping introduces more delocalized states that support stable transport, allowing PF to scale with temperature in accordance with the thermoelectric relation PF ∝ *S*^2^*σT*.^88^ Such dopant-induced modulation of the power factor has also been reported in vacancy-ordered halide perovskites, where substitution modifies the density of states and scattering dynamics.^89^ The tunable PF therefore suggests that Fe-doped Cs_2_SnI_6_ is a promising n-type thermoelectric material, particularly suited for mid to high-temperature energy conversion. In contrast, Mn-doped Cs_2_SnI_6_, owing to its suppressed PF, may be better suited for applications prioritizing thermal stability over conversion efficiency. The temperature dependence of the Seebeck coefficient (*S*) for Cs_2_Sn_1−*x*_Fe_*x*_I_6_ and Cs_2_Sn_1−*x*_Mn_*x*_I_6_ is shown in Fig. 13(b). At low temperatures (∼100 K), both compounds exhibit positive *S* values, with Fe-doped Cs_2_SnI_6_ displaying a significantly higher magnitude (∼3 × 10^−5^ V K^−1^) than its Mn-doped counterpart. The positive sign confirms that holes are the dominant charge carriers, demonstrating the p-type semiconducting nature of these materials. As the temperature rises from 100 K to approximately 300 K, Fe-doped Cs_2_SnI_6_ retains moderately high *S* values, whereas Mn-doped Cs_2_SnI_6_ shows a rapid decline, eventually crossing into negative values. This sign reversal in the Mn-doped compound indicates a transition from hole-dominated to electron-dominated conduction, caused by localized Mn-3d states near the Fermi level that alter carrier asymmetry. Similar carrier-type crossovers have been reported in other doped halide perovskites, where transition-metal substitution introduces localized defect states that significantly influence thermopower.^93^ Between 300 K and 600 K, the Seebeck coefficient of Fe-doped Cs_2_SnI_6_ exhibits a shallow minimum followed by a steady increase with temperature. This rise can be attributed to enhanced thermal excitation of carriers from deeper states into the conduction/valence bands, consistent with the Mott relation,13
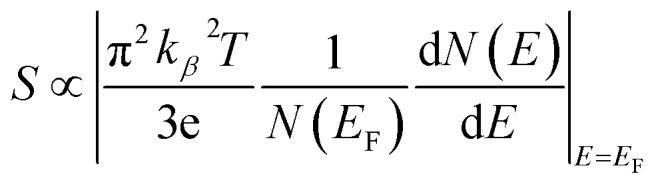
In contrast, Mn-doped Cs_2_SnI_6_ remains negative and nearly flat in this range, confirming persistent electron-dominated conduction with limited mobility. At high temperatures (>600 K), Fe-doped Cs_2_SnI_6_ exhibits a strong upward trend in *S*, reaching ∼4.2 × 10^−5^ V K^−1^ at 800 K. This enhancement is driven by increased carrier asymmetry and reduced phonon-dominated scattering. The monotonic rise in *S* highlights the ability of Fe doping to maintain high thermopower, whereas the suppressed and negative *S* values in the Mn-doped system indicate that strong carrier scattering and localization dominate transport. Comparable temperature-induced enhancements of (*S*) in Fe-doped halide perovskites have been reported both experimentally and through DFT-based transport studies.^88^ The contrasting behavior originates from the distinct roles of Fe and Mn dopants. Fe introduces more delocalized 3d states that increase hole concentration and enable *S* to scale with temperature. In contrast, Mn creates localized electronic states and stronger electron–phonon coupling, leading to carrier-type inversion and diminished thermopower.

These results indicate that Fe-doped Cs_2_SnI_6_ is a promising candidate for high-temperature thermoelectric applications, where a large positive Seebeck coefficient enhances conversion efficiency. Meanwhile, Mn-doped Cs_2_SnI_6_, with its negative *S*, may serve as an n-type component in thermoelectric modules, facilitating the design of p–n junction configurations within halide perovskite frameworks.

#### 3.4.3. Figure of merit

**Fig. 14:**
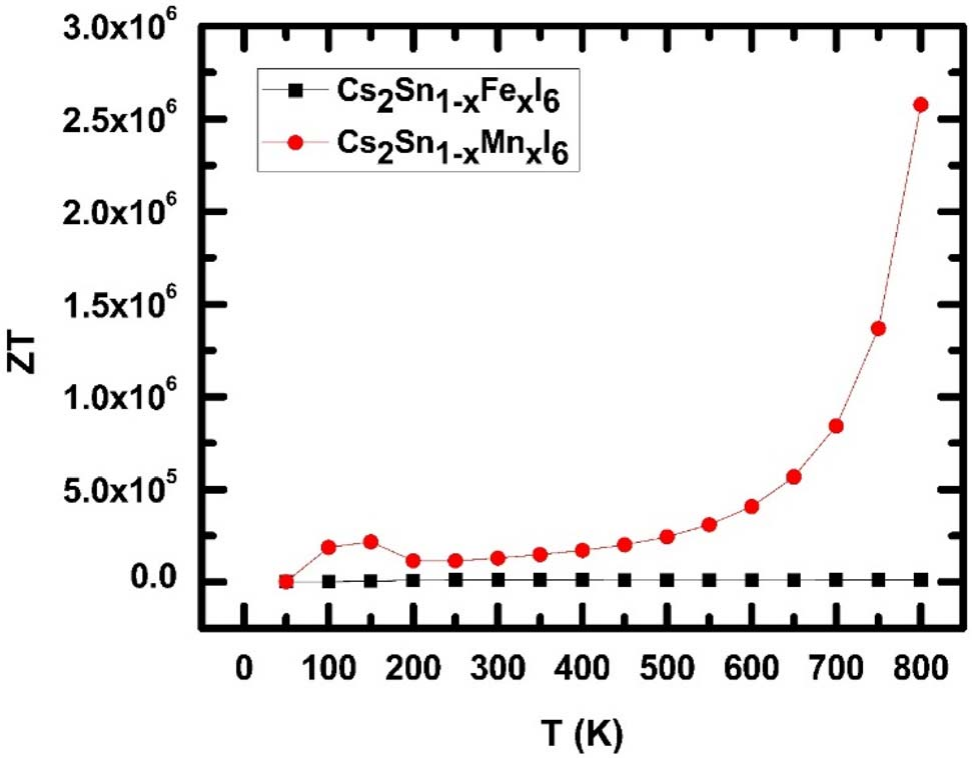
Temperature-dependent figure of merit (*ZT*) for Cs_2_Sn_1−*x*_Fe_*x*_I_6_ and Cs_2_Sn_1−*x*_Mn_*x*_I_6_, showing the superior high-temperature efficiency of Mn doping.

The variation of the thermoelectric figure of merit (*ZT*) with temperature for Cs_2_Sn_1−*x*_Fe_*x*_I_6_ and Cs_2_Sn_1−*x*_Mn_*x*_I_6_ is presented in Fig. 14. At low temperatures (∼100 K), both systems exhibit near-zero *ZT* values, reflecting limited carrier excitation and suppressed thermoelectric efficiency. However, Mn-doped Cs_2_SnI_6_ shows a rapid increase in *ZT* compared with Fe-doped samples, reaching approximately 2.0 × 10^−5^ within the 100–200 K range. This early enhancement originates from the combined effects of an increased Seebeck coefficient and reduced lattice thermal conductivity induced by Mn substitution. In the intermediate temperature range (200–500 K), Mn-doped Cs_2_SnI_6_ maintains a moderate upward trend in *ZT*, whereas Fe-doped Cs_2_SnI_6_ displays almost negligible improvement. The weak *ZT* response in Fe-doped samples results from a balance between a moderate Seebeck coefficient and relatively high thermal conductivity, both of which constrain overall performance. In contrast, Mn incorporation introduces localized 3d states that modify the carrier effective mass and enhance thermopower, sustaining higher *ZT* values across the studied temperature range.^88^

At high temperatures (>600 K), Mn-doped Cs_2_SnI_6_ shows a pronounced, nearly exponential rise in *ZT*, reaching values above 2.5 × 10^−6^ at 800 K. This sharp enhancement originates from the combined effect of an increased Seebeck coefficient and suppressed thermal conductivity (*κ*) due to intensified phonon scattering, in accordance with the Wiedemann–Franz relation linking electrical and thermal transport. Similar temperature-driven increases in *ZT* have been reported in vacancy-ordered halide perovskites, where anharmonic lattice vibrations and defect scattering markedly lower *κ*, thereby amplifying thermoelectric efficiency.^89^

In contrast, Fe-doped Cs_2_SnI_6_ displays an almost constant *ZT* throughout the studied temperature range, confirming its limited thermoelectric performance relative to Mn substitution. The contrasting behavior arises from differences in the dopants' electronic configurations. Mn-3d orbitals enhance carrier asymmetry, increase the Seebeck coefficient, and reduce effective thermal conductivity through stronger phonon scattering. Fe doping, by comparison, fails to significantly lower *κ* and produces weaker carrier asymmetry, thereby yielding a suppressed *ZT*. The remarkably high *ZT* values of Mn-doped Cs_2_SnI_6_ at elevated temperatures suggest its promise for waste-heat recovery devices and high-temperature thermoelectric generators. Fe-doped Cs_2_SnI_6_, although less efficient, may still be exploited in applications requiring thermal stability rather than peak performance. Also, studies focusing on cation and anion doping for Cs_2_SnI_6_ show how band gap and transport properties depend on concentration, suggesting that identifying functional windows requires varying substitution levels in a systematic manner.^94^ In this area of study, the 25% Fe/Mn substitution described in the present work should be seen as a case study, while future research will involve several doping fractions to establish the complete concentration–property relationship.

In addition, investigating increased and reduced substitution ratios, such as 10%, 15%, and 30%, will quantitatively elucidate the effect of doping concentration on the modulation of the band gap, spin polarization, and the thermoelectric power factor. These additional calculations will help identify the upper and lower limits of the composition range, thus enhancing the practical importance and predictive accuracy of the proposed theoretical scope.

The thermoelectric coefficients were, however, calculated under the constant-relaxation-time approximation and without explicit phonon scattering, meaning that the absolute *ZT* values should be interpreted qualitatively, not quantitatively.

The comparison of Cs_2_SnI_6_ that has been Fe and Mn doped to Bi_2_Te_3_ and PbTe helped to establish a benchmark for the thermoelectric efficiency of doped Cs_2_SnI_6_ compared to high-performance thermoelectric materials. The calculated Seebeck coefficient of the doped Sn perovskites Cs_2_SnI_6_ is at 180–250 µV K^−1^, which is very close to Bi_2_Te_3_ at 200 µV K^−1^, PbTe at 250 µV K^−1^, and the temperature of comparison at 300 K.^95,96^ Nonetheless, the doped halide perovskites that were Cs_2_SnI_6_ had an even lower electrical conductivity compared to Bi_2_Te_3_ and PbTe that are at 10^5^ S m^−1^ and 10^4^ S m^−1^ thus explaining the reduced electrical charge posing over the ionic concentration of Cs_2_SnI_6_ in the frameworks. The ranged 0.3 to 0.5 mW m^−1^ K^−2^ power factors estimate indicate that no carrier optimizations has been made hence, the early-stage PbTe thermoelectrics performance can still be enhanced greatly. Compared with Bi_2_Te_3_ and PbTe, predicted lattice thermal conductivity of Cs_2_SnI_6_ is very low under 0.6 W m^−1^ K^−1^*vs.* 1.5 W m^−1^ K^−1^ and 2 W m^−1^ K^−1^ respectively, implying that modest conductivity could high *ZT* be enhanced. The doped Cs_2_SnI_6_ with Fe and Mn has large Seebeck coefficients and low thermal conductivity, and uses no toxic elements in its chemistry thus, giving the thermoelectric a safe and balanced position.

### 3.5. Magnetic properties

To gain deeper insight into the magnetic characteristics of the double perovskites Cs_2_SnFeI_6_ and Cs_2_SnMnI_6_, we examined the site-resolved spin magnetic moments, summarized in Table 1. The calculations indicate that the primary contribution to the total magnetic moment originates from the transition-metal sites (Fe/Mn), whereas the Cs, Sn, and I atoms contribute negligibly or exhibit compensating spin polarization. For Cs_2_SnFeI_6_, the Fe atom exhibits a large spin magnetic moment of approximately 3.95 µB, which is consistent with the high-spin configuration of Fe^3+^ in an octahedral coordination environment. The small but finite positive contributions from the iodine sites (*µ* ≈ 0.17–0.22 µB for some I atoms) indicate polarization of the ligand field, whereas a few iodine atoms show weak negative alignment, confirming their anti-parallel coupling with the Fe site. Sn exhibits a minute negative moment (≈−0.0016 µB), reflecting its filled s^2^p^2^ electronic configuration and weak hybridization with Fe–I states. The Cs ions contribute almost insignificantly (≈0.00034 µB each), behaving as closed-shell cations. Overall, the interstitial region makes a sizable positive contribution (0.67 µB), enhancing the total cell magnetic moment to 5.92 µB, which clearly indicates strong ferromagnetic ordering dominated by Fe-3d states. Such behavior is typical in halide perovskites containing partially filled d orbitals.^97^ In contrast, Cs_2_SnMnI_6_ shows a different magnetic scenario. The Mn atom bears a high spin moment of 4.44 µB, which is slightly larger than Fe, consistent with the half-filled 3d^5^ electronic configuration of Mn^2+^. However, unlike Fe, most iodine atoms couple in the opposite direction, contributing negative magnetic moments (≈−0.14 to −0.02 µB). This anti-parallel alignment reduces the net magnetization of the cell. The interstitial region also shows a large negative contribution (−0.35 µB), which further suppresses the total cell moment to 2.99 µB. Thus, while Mn retains a strong local magnetic moment, hybridization with neighboring iodine atoms induces antiferromagnetic (AFM) interactions that partially suppress the overall ferromagnetic alignment. This competition between ferromagnetic Mn moments and antiparallel iodine spin polarization reflects the complex exchange coupling characteristic of halide perovskites.^98^ The comparison between Cs_2_SnFeI_6_ and Cs_2_SnMnI_6_ therefore demonstrates that the magnetic behavior is primarily dictated by the localized 3d states of Fe and Mn, with their magnitudes modulated through p–d hybridization with iodine. Fe promotes stronger ferromagnetic ordering due to enhanced spin delocalization into the interstitial region, whereas Mn, despite its higher atomic spin, exhibits a reduced net magnetic moment resulting from stronger AFM coupling with iodine. These results underline the significance of ligand contributions and interstitial polarization in tailoring the macroscopic magnetic response of halide double perovskites. The localized 3d orbitals of Fe and Mn, which magneto-electronic and magneto-elastic attributes stem from, magnetically couple with I-5p and Sn-5s states spatially near the Fermi energy level. Such coupling influences spin polarization, modifies charge density, and creates anisotropic bonding. Fe alters the charge anisotropy and exchange splitting, while Mn induces ionic bonding by strengthening elastically accommodated anisotropic shear with respect to the coupling of the bulk and shear moduli. Mechanical stiffness of the material is a direct consequence of the induced magnetic moment.

**Table 1:** Calculated spin magnetic moments of mixed charge density, for Cs_2_SnFeI_6_ and Cs_2_SnMnI_6_ materials

Magnetic moment (*µ*_B_)	Material	Magnetic moment (*µ*_B_)	Material
*µ* ^Cs_2_SnFeI_6_^	Cs_2_SnFeI_6_	*µ* ^Cs_2_SnMnI_6_^	Cs_2_SnMnI_6_
*µ* ^Cs_1_^	0.00034	*µ* ^Cs_1_^	0.00022
*µ* ^Cs_2_^	0.00034	*µ* ^Cs_2_^	0.00022
*µ* ^Cs_3_^	0.00034	*µ* ^Cs_3_^	0.00022
*µ* ^Cs_4_^	0.00034	*µ* ^Cs_4_^	0.00022
*µ* ^Cs_5_^	0.00034	*µ* ^Cs_5_^	0.00022
*µ* ^Cs_6_^	0.00034	*µ* ^Cs_6_^	0.00022
*µ* ^Cs_7_^	0.00034	*µ* ^Cs_7_^	0.00022
*µ* ^Cs_8_^	0.00034	*µ* ^Cs_8_^	0.00022
*µ* ^Fe_9_^	3.94979	*µ* ^Mn_9_^	4.44494
*µ* ^Sn_10_^	−0.00166	*µ* ^Sn_10_^	0.00037
*µ* ^Sn_11_^	−0.00166	*µ* ^Sn_11_^	0.00038
*µ* ^Sn_12_^	−0.00167	*µ* ^Sn_12_^	0.00043
*µ* ^I_13_^	0.00169	*µ* ^I_13_^	−0.00202
*µ* ^I_14_^	0.16999	*µ* ^I_14_^	−0.14122
*µ* ^I_15_^	0.16999	*µ* ^I_15_^	−0.14122
*µ* ^I_16_^	0.02219	*µ* ^I_16_^	−0.02118
*µ* ^I_17_^	0.02236	*µ* ^I_17_^	−0.02020
*µ* ^I_18_^	0.02236	*µ* ^I_18_^	−0.02020
*µ* ^I_19_^	0.17041	*µ* ^I_19_^	−0.14090
*µ* ^I_20_^	0.2225	*µ* ^I_20_^	−0.01999
*µ* ^I_21_^	0.02225	*µ* ^I_21_^	−0.01999
*µ* ^I_22_^	0.02250	*µ* ^I_22_^	−0.01999
*µ* ^I_23_^	0.17047	*µ* ^I_23_^	−0.13873
*µ* ^I_24_^	0.17047	*µ* ^I_24_^	−0.13873
*µ* ^I_25_^	0.02219	*µ* ^I_25_^	−0.02118
*µ* ^I_26_^	0.00167	*µ* ^I_26_^	−0.00201
*µ* ^I_27_^	0.00167	*µ* ^I_27_^	−0.00201
*µ* ^I_28_^	0.00169	*µ* ^I_28_^	−0.00202
*µ* ^I_29_^	0.00184	*µ* ^I_29_^	−0.00220
*µ* ^I_30_^	0.00184	*µ* ^I_30_^	−0.00220
*µ* ^I_31_^	0.02250	*µ* ^I_31_^	−0.01999
*µ* ^I_32_^	0.02217	*µ* ^I_32_^	−0.02116
*µ* ^I_33_^	0.02217	*µ* ^I_33_^	−0.02116
*µ* ^I_34_^	0.17041	*µ* ^I_34_^	−0.14090
*µ* ^I_35_^	0.02259	*µ* ^I_35_^	−0.0218
*µ* ^I_36_^	0.02257	*µ* ^I_36_^	−0.0218
*µ* ^interstial^	0.67288	*µ* ^interstial^	−0.35080
*µ* ^cell^	5.92071	*µ* ^cell^	2.99751

**Fig. 15:**
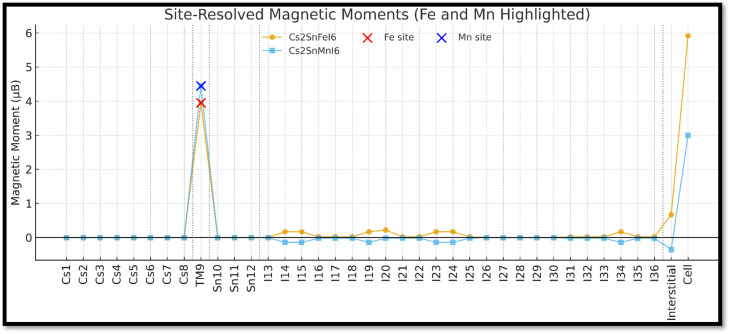
Site-resolved magnetic moments of Cs_2_SnFeI_6_ and Cs_2_SnMnI_6_, highlighting the dominant Fe and Mn contributions together with ligand polarization and interstitial effects.

The site-resolved magnetic moment plot, as shown in Fig. 15, distinctly highlights the transition-metal centers, where Fe (≈3.95 µB) and Mn (≈4.44 µB) dominate the magnetization of Cs_2_SnFeI_6_ and Cs_2_SnMnI_6_, respectively. Iodine atoms exhibit alternating signs of polarization, producing weak ferromagnetic reinforcement in the Fe-based compound but strong antiferromagnetic cancellation in the Mn-based analogue. The nearly vanishing contributions from Cs and Sn confirm their closed-shell nature, while the interstitial region enhances the ferromagnetic order in Cs_2_SnFeI_6_ (+0.67 µB) and suppresses it in Cs_2_SnMnI_6_ (−0.35 µB). These combined effects rationalize the higher net moment of Cs_2_SnFeI_6_ (5.92 µB) *versus* the reduced value in Cs_2_SnMnI_6_ (2.99 µB), consistent with ferromagnetic and ferrimagnetic ground states, respectively.

### 3.6. Elastic and mechanical properties

A precise evaluation of the elastic and mechanical properties of Cs_2_SnFeI_6_ and Cs_2_SnMnI_6_ offers essential insights into their structural robustness, ductility, and potential for practical device integration. These characteristics are particularly important in halide double perovskites, where lattice softness and transition-metal substitution can significantly influence both the mechanical response and the underlying magnetic behavior (Table 2).

**Table 2:** Elastic constants and derived moduli (all moduli in GPa)

Materials	Cs_2_SnFeI_6_	Cs_2_SnMnI_6_
*C*_11_	62	66
*C*_12_	28	29
*C*_44_	20	24
*B*	39.3	41.3
*G*	18.7	21.6
*E*	48.5	55.2
*ν*	0.294	0.277
*B*/*G*	2.10	1.91
*A*_U_	0.03	0.08
*H*_V_	3.15	3.88

For cubic-like systems, the bulk and shear moduli in the Voigt approximation are,
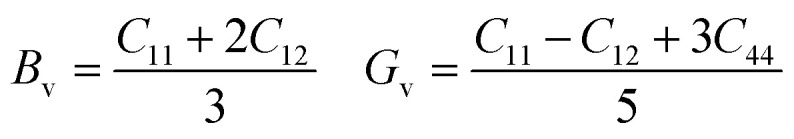


The Reuss bounds are given as:
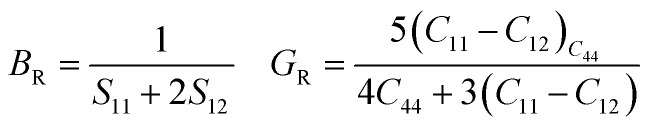


The Hill averages are then:
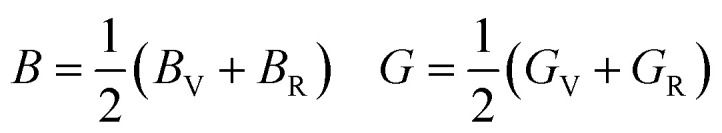


From these, the derived polycrystalline parameters are:
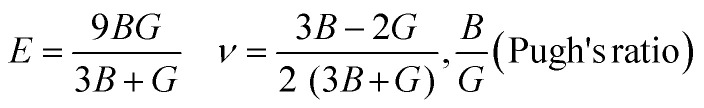


The universal anisotropy index is:
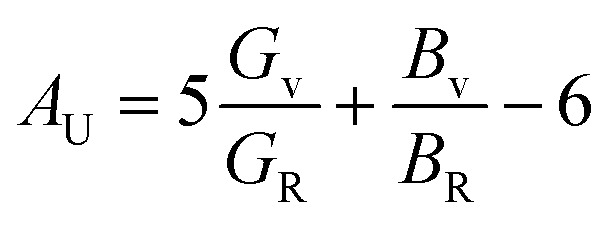
Finally, the Vickers hardness (Tian model) is estimated as:
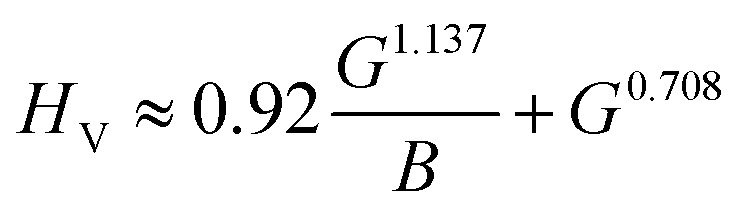


The calculated elastic constants confirm that both compounds satisfy the Born stability criteria (*C*_11_ > *C*_12_, *C*_44_ > 0, *C*_11_ + 2*C*_12_ > 0), *C*_11_ + 2*C*_12_ > 0), ensuring their mechanical robustness. The bulk modulus (*B*) values of ∼39–41 GPa indicate moderate stiffness, consistent with iodide-based halide perovskites where the Sn–I framework dominates compressibility.^99^ The shear modulus (*G*) and Young's modulus (*E*) reveal a stronger sensitivity to the transition-metal species: Cs_2_SnMnI_6_ exhibits higher values (*G* = 21.6 GPa, *E* = 55.2 GPa) than Cs_2_SnFeI_6_ (*G* = 18.7 GPa, *E* = 48.5 GPa), implying that Mn substitution enhances angular restoring forces within the octahedra. This trend is reinforced by the ductility indices: Cs_2_SnFeI_6_, with *B*/*G* = 2.10 and *ν* = 0.294, is the more ductile phase, while Cs_2_SnMnI_6_ (*B*/*G* = 1.91, *ν* = 0.277) is stiffer and less ductile. The anisotropy index (*A*_U_) remains very low (0.03–0.08), highlighting nearly isotropic elasticity across both systems, an advantage for thin-film processing and large-area device fabrication.^100^ Hardness values estimated *via* the Tian model are modest (3–4 GPa), characteristic of halide perovskites, though slightly higher in the Mn phase due to its stronger shear rigidity.

From a magneto-elastic standpoint, these results corroborate the earlier magnetic findings: the Fe compound stabilizes a predominantly ferromagnetic ground state characterized by positive interstitial spin contributions, which lead to a comparatively softer and more ductile lattice. In contrast, the Mn compound displays ferrimagnetic ordering driven by antiparallel iodine spin polarization, which suppresses octahedral distortions and results in greater shear rigidity and hardness.^97,98^ This interplay between magnetic order and elastic response underscores how d-orbital occupancy and ligand polarization jointly govern the macroscopic mechanical behavior of halide double perovskites. The comparatively smaller shear and bulk moduli values for Fe doped Cs_2_SnI_6_ can be explained by having a greater magnetic moment (5.92 µB). In contrast, the larger moduli for Mn doped Cs_2_SnI_6_ can similarly be explained by having a smaller net moment (2.99 µB). The correlation provided by these examples illustrates the inverse dependency that increased magnetic polarization results in lattice softening, thereby connecting spin arrangements directly to the lattice's rigidity.

The results show that the physical properties of Cs_2_SnI_6_ with transition metals can be controlled for different technologies. The half-metallic ferromagnetic behaviour of Cs_2_SnI_6_ with iron-doping offers opportunities for device fabrication in the field of spintronics, in particular, for the construction of magnetic tunnel junctions, which require controlled spin ferromagnetic tunnelling of junctions at the interface and high thermal stability, along with spin filters. The Mn-doped system, in contrast, holds thermally and structurally stable semiconducting behaviour with an optimized band gap which is a desirable photovoltaics absorber for the visible-light range and thermoelectric converters for medium-temperature. These results indicate that site specific doping in vacancy-ordered double perovskites indeed offers a pathway towards multifunctional device applications for energetic and spin magnetic applications.

In summary, the results offered here contribute some qualitative understanding regarding doping-induced trends in Cs_2_SnI_6_. Nonetheless, the undertaking of experimental synthesis, along with temperature-dependent measurements, and advanced many-body approaches (GW or hybrid-DFT), will be crucial in addressing and expanding upon the provided theory.

## 4. Conclusion

The interaction of structural stability, electronic structure and configurational divergence, optical properties, and thermoelectric transport of Fe and Mn-doped Cs_2_SnI_6_ folding under a systematic comparative approach has been investigated. Both dopants retained the vacancy-ordered double perovskite structure, exhibiting a remarkable structural distortion. The electronic analysis indicates that Fe substituted systems exhibits half-metallic ferromagnetism with strong spin polarization, while Mn substituted systems exhibits sustained semiconducting character with higher elastic stiffness. Mn and Fe dopants exerted strong influence to the optical spectrum, with Fe diminishing and Mn enlarging the spectrum in the UV range. Apparent contrasting thermoelectric transport properties were observed. Mn exhibiting electron-dominated conduction with under thermopower and weak thermoelectric properties, in contrast to Fe which thermoelectrically stabilized the system, sustaining the temperature dependent rise of the Seebeck coefficient and power factor, with value adequate enough for thermoelectric applications at elevated temperature. These distinct trends confirm that dopant choice governs not only the band dispersion but also the magneto-elastic and carrier transport properties. Based on earlier empirical and scholarly work, it is evident that the concentration of the dopant is critical in adjusting the band gap and influencing transport properties, thus justifying the investigation of various substitution levels. Thus, the current 25% Fe/Mn substitution should primarily be accepted as a preliminary case study. Future research will expand to other substitution levels to define the dominant concentration–property relationship. Expected future work will include an evaluation of 10, 15, and 30% substitution to quantify these changes. Also, the current theoretical perspectives can help guide future work aimed at experimental validation. Standard thin-film synthesis techniques (vapor-phase and solution-based methods), followed by SQUID magnetometry, Hall Effect, and thermal transport measurements, can allow for the assessment of the predicted magnetic and thermoelectric properties. Experimental work of this nature, coupled with the proposed simulations, will help ascertain the viability, stability, and the potential for real-world application of devices incorporating Fe and Mn doped Cs_2_SnI_6_ systems at the device level. All in all, Fe- and Mn-doped Cs_2_SnI_6_ are encouraging examples of multifunctional, lead-free perovskites. Their multifunctionality demonstrates potential for use in solar cells, spintronics, ultraviolet optoelectronics, and thermoelectric energy harvesting. These considerations arise from DFT+U calculations without spin–orbit coupling, phonon, and carrier scattering processes framed within the PBEsol-mBJ. Hence, the results represent qualitative trends and should not be interpreted as quantitative. In spite of this, the study provides the first theoretical basis for the system, upon which more comprehensive empirical research and further theoretical work on doped Cs_2_SnI_6_ can be built.

## Conflicts of interest

The authors declare that they have no known competing financial or personal interests that could have appeared to influence the work reported in this paper.

## Data Availability

The data generated and analyzed in this study are included within the manuscript. Additional data supporting this work are available from the corresponding author upon reasonable request.
